# Mechanical properties of artificially structured soil and Binary-medium-based constitutive model under undrained conditions

**DOI:** 10.1371/journal.pone.0296441

**Published:** 2024-01-03

**Authors:** Yizhi Li, Enlong Liu, Miao He

**Affiliations:** State Key Laboratory of Hydraulics and Mountain River Engineering, College of Water Resources and Hydropower, Sichuan University, Chengdu, China; Instituto Tecnologico de Aeronautica, BRAZIL

## Abstract

To investigate the mechanical properties and constitutive models of structured soil under undrained conditions, triaxial compression tests on initially anisotropic structured soil, isotropic structured soil, and remolded soil were conducted under consolidation undrained conditions at confining pressures of 25, 50, 100, and 200 kPa, respectively. The results demonstrate that the samples of structured soils with strong structural characteristics have an obvious yield strength when the consolidation stress is low. At this time, the pore water pressure in structured soils increases at the beginning of loading. As the axial strain increasing, it turns to reduce. When failure, the samples have obvious shear band. With the consolidation stress increases, the mechanical properties and deformation mechanism of structured soils are near to the remolded soil. Combining the Binary-medium theory with the analysis and discussion of the mechanical properties and deformation mechanisms of structured soil, the rationality of the corresponding Binary-medium model was verified, which shows that the constitutive model can reflect the characteristics of dilatancy and strain softening, volumetric contraction and strain hardening under the conditions of low and high confining pressure respectively. At the same time, the constitutive model can also reflect the differences in the stress-strain characteristics of the two structural soils caused by the structural differences. In general, the results agree with the experiment relative well.

## 1 Introduction

Natural soils are generally inhomogeneous structural materials and are a widespread geotechnical material [1–6]. However, in practical engineering, due to a lack of understanding of the characteristics of natural soils, especially its mechanical properties, numerous engineering issues have arisen, and accidents that endanger the lives and property of the public have occurred [7, 8]. The structural nature of soil is an important mechanical property [9]. Considering the influence of structure on the mechanical properties of natural soils [10–12]. For example, the microscopic properties of soils generally refer to characteristics that lie between the macroscopic and microscopic scales, such as strain localization (shear) and crack propagation [13]. Therefore, research on structured soils is of significant importance. The constitutive model of soil has always been one of the hot and focal issues in the field of geotechnical engineering. It serves as the foundation for engineering problem analysis and numerical simulation. The accuracy of the constitutive model directly affects the correctness of the analysis results [14]. Previous research on remolded soils has made significant achievements. Various constitutive models for soils, such as the Cambridge model, Duncan-Chang model, and others, have been proposed and widely applied [15, 16]. Moreover, remarkable achievements have also been made in the research on the microstructure of remolded soils [17]. Natural soils typically exhibit structural characteristics. The presence of soil structure leads to significant differences in the mechanical properties of natural structured soils and remolded soils [1, 4, 5, 18–20].

For the study of constitutive models for such structured soils, He et al. proposed a sub-plastic constitutive model for structured soils. This model can describe the mechanical behavior of structured soils and remolded soils under different stress levels during normal consolidation and overconsolidation [21]. Liu et al. proposed a constitutive model to simulate the stress-strain characteristics of over consolidated structured soils [4]. The model also incorporates the concept of sub-loading plasticity to avoid overestimating the peak shear strength of structured soils in overconsolidated states. It evaluates the performance of the proposed model in describing the mechanical behavior of structured soils with different initial overconsolidation ratios. In the experimental study of structured soils, Liu et al. proposed a constitutive model that can explain the hydraulic response of unsaturated structured soils. This model extends the volumetric strain equation to a three-dimensional stress state by introducing a modified Cam-Clay model. It was validated using experimental data from structured soils and remolded soils [4]. Lin et al. explained the mechanism of the effect of structural characteristics on the mechanical evolution from a micromechanical perspective. They combined Discrete Element Method (DEM) with laboratory experiments to study the structural characteristics of natural undisturbed soil. The proposed DEM simulation method can be applied to structured soils and provide better guidance for engineering practice [22]. The binary medium model was put forward by Shen [23] and Liu et al. [24], which can be employed to model the structured geological materials. In the binary medium model, the microscopic units can indeed be divided into the bonded elements and frictional elements, and thus the structured geotechnical materials can then be abstracted as a binary medium composed of these two kinds of units. In this case, the cemented element can be described by the elastic-brittle constitutive model, while the frictional element can be described by the elastic-plastic constitutive model. This division is based on the understanding of the microscopic changes in the material during the process of loading. During the loading process, the elastic-brittle elements gradually break down and transform into elastic-plastic elements. This process reflects the complex nature of the material as it transitions from elastic to plastic deformation. In contrast, the theory of composites is mainly used to describe isotropic materials, such as resin matrix composites or metal composites. In composite material theory, it is usually assumed that all components are microscopically uniformly distributed and that their properties can be obtained in some average way. In addition, the volume fraction in the composite material is constant upon loading, but in the binary medium model developed here, the bonded element will break up and transform into frictional element.

In order to further investigate the mechanical characteristics and deformation mechanisms of structured soils, we conducted conventional triaxial compression tests on structured soils under undrained conditions. Based on the test results and in combination with the derivation and computational results of the Binary-medium model, we verified the rationality of the corresponding Binary-medium model. Specifically, three types of specimens: initially stress anisotropic structured soil, initially stress isotropic structured soil, and remolded soil were prepared. Undrained triaxial compression tests on these specimens under five different consolidation pressures: 25 kPa, 50 kPa, 100 kPa, and 200 kPa were conducted. Based on the test results, the relevant computational parameters of the incremental binary-medium model are determined and the computed results with the experimental results are also compared to validate the model.

## 2 Constitutive model development

### 2.1 Derivation of the Binary-medium constitutive model

According to the fundamental theory of the binary-medium model [23], the average stress {*σ*} and average strain {*ε*} can be expressed in the following form:

$$\{\sigma \}=(1-\lambda ){\left\{\sigma \right\}}_{b}+\lambda {\left\{\sigma \right\}}_{f}$$ (1)

$$\{\varepsilon \}=(1-\lambda ){\left\{\varepsilon \right\}}_{b}+\lambda {\left\{\varepsilon \right\}}_{f}$$ (2)

where *λ* represents the volumetric damage strain of the structured soil; {*σ*}_*b*_, {*σ*}_*f*_, {*ε*}_*b*_, {*ε*}_*f*_, denote the local stress and local strain of the brittle-elastic component and elasto-plastic component, respectively. We assume that the volumetric damage strain is a function of the strain and it can be expressed in the following form:

λ=f({ε}) (3)

Based on Eqs (1)–(3), the incremental formulas for average stress and average strain can be derived, as shown in Eqs (4)–(5).


$$\left\{\Delta \sigma \}=(1-\lambda^{o}){\left\{\Delta \sigma \right\}}_{b}+\lambda^{o}{\left\{\Delta \sigma \right\}}_{f}+\Delta \lambda ({\left\{\sigma \right\}}^{o}{}_{f}-{\left\{\sigma \right\}}^{o}{}_{b}\right)$$ (4)

$$\left\{\Delta \varepsilon \}=(1-\lambda^{o}){\left\{\Delta \varepsilon \right\}}_{b}+\lambda^{o}{\left\{\Delta \varepsilon \right\}}_{f}+\Delta \lambda ({\left\{\varepsilon \right\}}^{o}{}_{f}-{\left\{\varepsilon \right\}}^{o}{}_{b}\right)$$ (5)

In which {Δ*σ*}, {Δ*ε*}: the calculated increments of average stress and average strain. {*σ*}^*o*^_*b*_, {*σ*}^*o*^_*f*_, {*ε*}^*o*^_*b*_, {*ε*}^*o*^_*f*_: Local stress and strain of the brittle-elastic components and elastic-plastic components before the increment calculation. *λ*^0^: Volume damage rate at the beginning of the current increment step. Δ*λ*: Increment of volume damage rate. {Δ*σ*}_*b*_, {Δ*σ*}_*f*_, {Δ*ε*}_*b*_, {Δ*ε*}_*f*_: Local stress and strain increments of the brittle-elastic components and elastic-plastic components.

The stress increments of the brittle-elastic components and elastic-plastic components, denoted as [D]_*b*_ and [D]_*f*_ respectively, satisfy the following equations:

$${\left\{\Delta \sigma \right\}}_{b}={\left[D\right]}_{b}{\left\{\Delta \varepsilon \right\}}_{b}$$ (6)

Δσ=fDepfΔεf (7)

Eq (5) can be rearranged as follows:

$${\left\{\Delta \varepsilon \right\}}_{f}=\frac{1}{\lambda^{o}}\left\{\{\Delta \varepsilon \}-(1-\lambda^{o}){\left\{\Delta \varepsilon \right\}}_{b}-\Delta \lambda \left[{\left\{\varepsilon \right\}}^{o}{}_{f}-{\left\{\varepsilon \right\}}^{o}{}_{b}\right]\right\}$$ (8)

Substituting Eq (8) into Eqs (6) and (7), we obtain:

$${\left\{\Delta \sigma \right\}}_{f}=\frac{1}{\lambda^{o}}{\left[D\right]}_{f}\left\{\{\Delta \varepsilon \}-(1-\lambda^{o}){\left\{\Delta \varepsilon \right\}}_{b}-\Delta \lambda \left[{\left\{\varepsilon \right\}}^{o}{}_{f}-{\left\{\varepsilon \right\}}^{o}{}_{b}\right]\right\}$$ (9)

Substituting Eq (9) into Eq (4), we obtain:

$$\{\Delta \sigma \}=(1-\lambda^{o})\left\{{\left[D\right]}_{b}-{\left[D\right]}_{f}\right\}{\left\{\Delta \varepsilon \right\}}_{b}+{\left[D\right]}_{f}\{\Delta \varepsilon \}-\Delta \lambda {\left[D\right]}_{f}\left\{{\left\{\varepsilon \right\}}^{o}{}_{f}-{\left\{\varepsilon \right\}}^{o}{}_{b}\right\}+\Delta \lambda \left\{{\left\{\sigma \right\}}_{f}^{o}-{\left\{\sigma \right\}}_{b}^{o}\right\}$$ (10)

To establish the relationship between local strain and average strain for the brittle-elastic components, the local strain matrix [*C*] is introduced, which is expressed as follows:

$${\left\{\varepsilon \right\}}_{b}=[C]\{\varepsilon \}$$ (11)

The incremental form expression of Eq (11) is as follows:

$${\left\{\Delta \varepsilon \right\}}_{b}={\left[C\right]}^{o}\{\Delta \varepsilon \}+[\Delta C]{\left\{\varepsilon \right\}}^{o}$$ (12)

where [*C*]^*o*^ represents the local strain matrix at the beginning of the current increment step, and [*ΔC*] represents the increment of the local strain matrix. By substituting Eq (12) into Eq (10) and rearranging, the general expression for stress increment is obtained as follows:

$$\begin{array}{c} \{\Delta \sigma \}=\left\{{\left[D\right]}_{f}+(1-\lambda^{o})\{{\left[D\right]}_{b}-{\left[D\right]}_{f}\}{\left[C\right]}^{o}\right\}\{\Delta \varepsilon \}+(1-\lambda^{o})\{{\left[D\right]}_{b}-{\left[D\right]}_{f}\}[\Delta C]{\left\{\varepsilon \right\}}^{o}\\ -\Delta \lambda {\left[D\right]}_{f}\left\{{\left\{\varepsilon \right\}}_{f}^{o}-{\left\{\varepsilon \right\}}_{b}^{o}\right\}+\Delta \lambda \left\{{\left\{\sigma \right\}}_{f}^{o}-{\left\{\sigma \right\}}_{b}^{o}\right\} \end{array}$$ (13)

Eqs (1) and (3) can be rewritten in the following forms:

$${\left\{\sigma \right\}}_{f}^{o}=\frac{{\left\{\sigma \right\}}^{o}-(1-\lambda^{o}){\left\{\sigma \right\}}_{b}^{o}}{\lambda^{o}}$$ (14)

$${\left\{\varepsilon \right\}}_{f}^{o}=\frac{{\left\{\varepsilon \right\}}^{o}-(1-\lambda^{o}){\left\{\varepsilon \right\}}_{b}^{o}}{\lambda^{o}}$$ (15)

By substituting Eqs (14) and (15) into Eq (13) and rearranging, the expression for the stress increment under general stress conditions can be obtained as follows:

$$\begin{array}{c} \{\Delta \sigma \}=\left\{{\left[D\right]}_{f}+(1-\lambda^{o})\{{\left[D\right]}_{b}-{\left[D\right]}_{f}\}{\left[C\right]}^{o}\right\}\{\Delta \varepsilon \}+(1-\lambda^{o})\{{\left[D\right]}_{b}-{\left[D\right]}_{f}\}[\Delta C]{\left\{\varepsilon \right\}}^{o}\\ -\frac{\Delta \lambda }{\lambda^{o}}{\left[D\right]}_{f}\left\{{\left\{\varepsilon \right\}}^{o}-{\left\{\varepsilon \right\}}_{b}^{o}\right\}+\frac{\Delta \lambda }{\lambda^{o}}\left\{{\left\{\sigma \right\}}^{o}-{\left\{\sigma \right\}}_{b}^{o}\right\} \end{array}$$ (16)

Without considering the structural damage in the specimen before the onset of shearing, at the initial moment: *λ*^0^ = 0, {*ε*}^*o*^ = 0, {*ε*}^*o*^_*b*_ = 0, {*ε*}^*o*^_*f*_ = 0. By substituting these initial conditions into Eq (13), the expression for the stress increment at the initial state can be obtained as follows:

$$\{\Delta \sigma \}=\left\{{\left[D\right]}_{f}+(1-\lambda^{o})\{{\left[D\right]}_{b}-{\left[D\right]}_{f}\}{\left[C\right]}^{o}\right\}\{\Delta \varepsilon \}+(1-\lambda^{o})\{{\left[D\right]}_{b}-{\left[D\right]}_{f}\}[\Delta C]{\left\{\varepsilon \right\}}^{o}$$ (17)

### 2.2 Constitutive relationship and parameter determination for brittle-elastic components

#### 2.2.1 Constitutive relationship for Brittle-elastic components

In general, natural soils possess structural characteristics. However, due to factors such as deposition conditions, natural soils often exhibit characteristics of anisotropy transversely and isotropy in other directions. In this experiment, the initial stress anisotropic structural soil was subjected to axial compressive loading during the curing process. Due to the lateral rigid constraint provided by the triaxial membrane, the prepared structural soil exhibited structural characteristics and showed an approximate isotropy transversely similar to natural soils. Based on the brittle-elastic components constitutive relationship in the initial stress anisotropic structured soil can be expressed as [24]:

$${\left\{\Delta \sigma \right\}}_{b}={\left[D\right]}_{b}{\left\{\Delta \varepsilon \right\}}_{b}$$ (18)

We represent the vertical direction as the z-axis, while the x-axis and y-axis lie in the horizontal plane perpendicular to the z-axis. The Eq (18) can be expanded as follows:

$${\left\{\begin{array}{c} \Delta \sigma_{x}\\ \Delta \sigma_{y}\\ \Delta \sigma_{z}\\ \Delta \tau_{yz}\\ \Delta \tau_{zx}\\ \Delta \tau_{xy} \end{array}\right\}}_{b}={\left[\begin{array}{cccccc} D_{11} & D_{12} & D_{13} & 0 & 0 & 0\\ D_{12} & D_{11} & D_{13} & 0 & 0 & 0\\ D_{13} & D_{13} & D_{33} & 0 & 0 & 0\\ 0 & 0 & 0 & D_{44} & 0 & 0\\ 0 & 0 & 0 & 0 & D_{44} & 0\\ 0 & 0 & 0 & 0 & 0 & \frac{\left(D_{11}-D_{12}\right)}{2} \end{array}\right]}_{b}{\left\{\begin{array}{c} \Delta \varepsilon_{x}\\ \Delta \varepsilon_{y}\\ \Delta \varepsilon_{z}\\ \Delta \varepsilon_{yz}\\ \Delta \varepsilon_{zx}\\ \Delta \varepsilon_{xy} \end{array}\right\}}_{b}$$ (19)

During the initial stage of load application, when the load is relatively small, the brittle-elastic components primarily bear the load in the structural soil. Therefore, the five mechanical parameters in the Eq (19): D_11_, D_12_, D_13_, D_33_, and D_44_ need to be determined based on the deviator stress-axial strain curve of the initial anisotropic structural soil under small deformation conditions. In particular, the isotropic structured soil tested in this study was prepared as a special case of the initially anisotropic structured soil. It exhibits the same mechanical properties in all directions, thus Eq (19) can also be used to represent the constitutive relationship of isotropic structured soil.

#### 2.2.2 Determination of parameters for the brittle-elastic constitutive model under triaxial stress state

Under conventional triaxial stress conditions, the Eq (19) can be simplified to:

$${\left\{\begin{array}{c} \Delta \sigma_{1}\\ \Delta \sigma_{3} \end{array}\right\}}_{b}=\frac{E_{vb}}{(1-\nu_{hhb})E_{vb}-2\nu_{vhb}^{2}E_{hb}}\left[\begin{array}{cc} (1-\nu_{hhb})E_{vb} & 2\nu_{vhb}E_{hb}\\ \nu_{vhb}E_{hb} & E_{hb} \end{array}\right]{\left\{\begin{array}{c} \Delta \varepsilon_{1}\\ \Delta \varepsilon_{3} \end{array}\right\}}_{b}$$ (20)

In Eq (20), *E*_*vb*_ and *E*_*hb*_ represent the elastic modulus of the brittle-elastic component in the vertical and horizontal directions, respectively. *v*_*vhb*_ and *v*_*hhb*_ represent the Poisson’s ratios of the brittle-elastic component in the vertical and horizontal directions, respectively. The parameter determination process of the brittle-elastic component reveals that its stiffness matrix is a constant value. When the axial strain is small, the volume damage of the structural soil is negligible. Therefore, under small strain conditions, the elastic modulus and Poisson’s ratio of the brittle-elastic component can be considered very close to the secant modulus and Poisson’s ratio of the sample. Assuming that the secant modulus and Poisson’s ratio of the isotropic structural soil are the same as the secant modulus and Poisson’s ratio of the initial anisotropic structural soil in the horizontal plane, the secant modulus and Poisson’s ratio of the brittle-elastic component can be determined through iterative calculations. The specific numerical values are shown in Table 1.

**Table 1:** Summary of elastic modulus and poisson’s ratio of brittle-elastic components under incremental theory.

Type	Confining Pressure/kPa	Evb /kPa	Ehb /kPa	*v*_*vhb*_	*v*_*hhb*_
Initial Anisotropic Structural Soil	25	16248	15145	0.381	0.406
	50	23949	22919	0.192	0.240
	100	31050	30050	0.105	0.205
	200	37793	36970	0.085	0.166
Isotropic Structural Soil	25	15145	15145	0.406	0.406
	50	22919	22919	0.240	0.240
	100	30050	30050	0.205	0.205
	200	36970	36970	0.166	0.166

### 2.3 Constitutive relationship and parameter determination for elastic-plastic components

#### 2.3.1 Constitutive relationship for elastic-plastic components

The constitutive model of structured soil utilizes the Lade-Duncan model [25] for analysis and computation [6, 26]. In the elastic-plastic constitutive model, when the soil reaches the plastic state, its strain can be divided into elastic strain and plastic strain components.

The constitutive model relationship for the elastic-plastic components can be expressed as follows:

$${\left\{\Delta \sigma \right\}}_{f}={\left[D^{ep}\right]}_{f}{\left\{\Delta \varepsilon \right\}}_{f}$$ (21)

where [*D*^*ep*^]_*f*_ represents the elastic-plastic stiffness matrix, which can be expressed in the following form:

$${\left[D^{ep}\right]}_{f}=\left({\left[D^{e}\right]}_{f}-\frac{{\left[D^{e}\right]}_{f}\left\{\frac{dg}{d\sigma }\right\}{\left\{\frac{df}{d\sigma }\right\}}^{T}{\left[D^{e}\right]}_{f}}{-\frac{\partial f}{\partial H}{\left\{\frac{\partial H}{\partial \varepsilon^{p}}\right\}}^{T}\left\{\frac{\partial g}{\partial \sigma }\right\}+\left\{\frac{\partial f}{\partial \sigma }\right\}{\left[D^{e}\right]}_{f}\left\{\frac{\partial g}{\partial \sigma }\right\}}\right)={\left[D^{e}\right]}_{f}-{\left[D^{p}\right]}_{f}$$ (22)

(1) Elastic Strain

The incremental elastic strain is determined by Hooke’s Law:

$${\left\{\Delta \varepsilon^{e}\right\}}_{f}={\left[D^{e}\right]}_{{}_{f}}^{-1}{\left\{\Delta \sigma^{e}\right\}}_{f}$$ (23)

In Eq (23), the elastic stiffness matrix is given by:

$${\left[D^{e}\right]}_{f}=\frac{E_{f}(1-\nu_{f})}{\left(1+\nu_{f})(1-2\nu_{f}\right)}\left[\begin{array}{cccccc} 1 & \frac{\nu_{f}}{1-\nu_{f}} & \frac{\nu_{f}}{1-\nu_{f}} & 0 & 0 & 0\\ \frac{\nu_{f}}{1-\nu_{f}} & 1 & \frac{\nu_{f}}{1-\nu_{f}} & 0 & 0 & 0\\ \frac{\nu_{f}}{1-\nu_{f}} & \frac{\nu_{f}}{1-\nu_{f}} & 1 & 0 & 0 & 0\\ 0 & 0 & 0 & \frac{1-2\nu_{f}}{2(1-\nu_{f})} & 0 & 0\\ 0 & 0 & 0 & 0 & \frac{1-2\nu_{f}}{2(1-\nu_{f})} & 0\\ 0 & 0 & 0 & 0 & 0 & \frac{1-2\nu_{f}}{2(1-\nu_{f})} \end{array}\right]$$ (24)

In Eq (24), *E*_*f*_ and *v*_*f*_ represent the secant modulus of elasticity and secant Poisson’s ratio of the elastic-plastic components, respectively. Considering that there are no brittle-elastic components in the reconstituted soil, the values of *E*_*f*_ and *v*_*f*_ can be determined through conventional triaxial compression tests on the reconstituted soil.

(2)Plastic Strain.

The Lade-Duncan failure criterion can be expressed as follows:

$$f_{1}=I_{1}^{3}/I_{3}=K_{f}$$ (25)

The yield function of this model can be represented as:

$$f=I_{1}^{3}/I_{3}=K$$ (26)

The plastic potential function is defined as follows:

$$g=I_{1}^{3}-K_{2}I_{3}$$ (27)

In Eq (27), *f* represents the stress level, *I*_1_ represents the first stress invariant, and *I*_3_ represents the third stress invariant. Then, by taking the derivative of Eq (27), we obtain the relationship between the plastic strain increment and the stress components, which can be expressed as:

ΔεxpΔεypΔεzpΔεyzpΔεzxpΔεyzpf=dϑ⋅K23I12K2−σyσ+zτxy23I12K2−σzσx+τzx23I12K2−σxσy+τxy22σxτyz−2τxyτzx2σyτzx−2τxyτyz2σzτxy−2τyzτzxf (28)

In Eq (28), *dϑ* represents the absolute magnitude of the plastic strain increment, and *K*_2_ represents the relative magnitude of the plastic strain increment.

#### 2.3.2 Determination of parameters for the elastic-plastic constitutive model under triaxial stress state

The constitutive relationship of the elastic-plastic component under conventional triaxial stress state can be expressed as follows:

$${\left\{\begin{array}{c} \Delta \sigma_{1}\\ \Delta \sigma_{3} \end{array}\right\}}_{f}=\left[D^{ep}\right]{\left\{\begin{array}{c} \Delta \varepsilon_{1}\\ \Delta \varepsilon_{3} \end{array}\right\}}_{f}$$ (29)

(1) Calculation of Elastic Parameters

The parameters *E*_*f*_ and *v*_*f*_ in the Eq (31) are determined using the method based on the Duncan-Chang model. The expressions for their calculation are as follows:

$$E_{f}=Kp_{a}{\left(\frac{\sigma_{3}}{p_{a}}\right)}^{n}{\left[1-\frac{R_{f}(\sigma_{1}-\sigma_{3})(1-\text{sin}\;\phi )}{2c\;\text{cos}\;\phi +2\sigma_{3}\;\text{sin}\;\phi }\right]}^{2}$$ (30)

$$\nu_{f}=\frac{G-F\;\text{lg}(\sigma_{3}/p_{a})}{{\left\{1-\frac{D(\sigma_{1}-\sigma_{3})}{Kp_{a}{\left(\frac{\sigma_{3}}{p_{a}}\right)}^{n}\left[1-\frac{R_{f}(\sigma_{1}-\sigma_{33})(1-\text{sin}\;\phi )}{2c\;\text{cos}\;\phi +2\sigma_{3}\;\text{sin}\;\phi }\right]}\right\}}^{2}}$$ (31)

In the equation, *K*, *n*, and *R*_*f*_ are material constants, while *c* and *φ* represent the cohesion and internal friction angle determined from triaxial compression tests on remolded soil. *p*_a_ denotes the standard atmospheric pressure. *G*, *F*, and *D* are also material constants, totaling 8 parameters in total. The method for determining the mechanical parameters of the elastic-plastic components is same as the Duncan-Chang nonlinear elastic constitutive model.

The current experiment is a triaxial compression test under undrained consolidation conditions, making it difficult to calculate the parameters of the Lade-Duncan model directly. However, the test specimen’s material and preparation method are the same as those described in the reference [6]. Therefore, the parameters mentioned above can be determined based on the findings of that reference. Please refer to Table 2 for the specific numerical values.

**Table 2:** Duncan-Chang model.

Parameter	*K*	*n*	*R*_*f*_	*c*	*φ*	*G*	*F*	*D*
Value	88.797	0.3425	0.95	0	32.063°	0.242	0.313	0.0113

(2) Calculation of plasticity-related parameters

Let the ratio of $\Delta \varepsilon_{x}^{p}$ to $\Delta \varepsilon_{z}^{p}$ be *v*^*p*^. Then, from the Eq (28), we can deduce the following expressions:

$$\nu^{p}=-\frac{\frac{3I_{1}^{2}}{K_{2}}-\sigma_{y}\sigma_{z}+\tau_{yz}^{2}}{\frac{3I_{1}^{2}}{K_{2}}-\sigma_{x}\sigma y+\tau_{xy}^{2}}$$ (32)

In the Eq (32), *v*^*p*^ represents the plastic Poisson’s ratio, which characterizes the ratio between the lateral plastic strain increment and the vertical plastic strain increment at failure. In the consolidated drained conditions of the conventional triaxial compression test, the specimen is in a principal stress state, where σ_*x*_ = σ_*y*_ = σ_3_, σ_z_ = σ_1_, τ_ij_ = 0 hold. By utilizing Eq (33), the expression of *K*_2_ in terms of the deviatoric stress (σ_1_ -σ_3_) can be obtained:

$$K_{2}=\frac{3{\left[\left(\sigma_{1}-\sigma_{3}\right)+3\sigma_{3}\right]}^{2}(1+\nu^{p})}{\sigma_{3}\left[\left(\sigma_{1}-\sigma_{3}\right)+\sigma_{3}(1+\nu^{p})\right]}$$ (33)

The value of *v*^*p*^ in the equation is determined based on the relationship between (σ_1_ -σ_3_), ε_1_, ε_3_, and ε_*v*_ obtained from conventional consolidated drained triaxial tests. According to Eq (33), it can be observed that *K*_2_ is determined for a given stress level. Therefore, the relationship between *K*_2_ and the stress level *f* can be expressed as:

$$K_{2}=Af+27\left(1-A\right)$$ (34)

The triaxial consolidated drained tests mentioned in the reference [6] indicate that *K*_2_ is directly proportional to *f* and is independent of the confining pressure *σ*_3_. Furthermore, it can be deduced that the numerical value of *A* in the expression for *K*_2_ = *Af* + 27(1 − *A*) is 0.3525.

The strain hardening law can be expressed as follows:

$$f=F(W_{p})$$ (35)

In Eq (35), the relationship between stress level *f* and plastic work *W*_*P*_ based on various compression consolidation drainage tests under different confining pressures can be expressed as follows:

$$f-f_{t}=\frac{W_{p}}{\alpha^{\prime }+\beta^{\prime }W_{p}}$$ (36)

In Eq (36), *f*_t_ represents the intersection point of the extrapolated *f*~*W*_*p*_ curve obtained at different confining pressures *σ*_3_. *α’* and *β’* are experimental parameters that vary with the confining pressure *σ*_3_.

The Eq (26) can be rewritten as a function of the deviatoric stress and the minimum principal stress:

$$f=\frac{{\left[\left(\sigma_{1}-\sigma_{3}\right)+3\sigma_{3}\right]}^{3}}{\left[\left(\sigma_{1}-\sigma_{3}\right)+\sigma_{3}\right]\sigma_{3}{}^{2}}$$ (37)

From Eq (37), it can be observed that as the deviatoric stress (σ_1_-σ_3_) approaches zero, *f* approaches 27, which implies that *f*_t_ in Eq (34) is 27. This holds true for conventional triaxial consolidated-undrained tests.


$$W_{p}=\int \sigma_{ij}d\varepsilon_{ij}^{p}$$ (38)

Therefore, the Eq (38) can be written as:

$$W_{p}=\int \left(\sigma_{1}d\varepsilon_{1}^{p}+2\sigma_{3}d\varepsilon_{3}^{p}\right)=\int \left(\sigma_{1}-\sigma_{3}\right)d\varepsilon_{1}^{p}+\int \sigma_{3}\left(d\varepsilon_{1}^{p}+d\varepsilon_{3}^{p}\right)$$ (39)

Substituting *dε*^*p*^ = *dε* − *dε*^*e*^ into the Eq (39), we get the following expression:

$$W_{p}=\int \left(\sigma_{1}-\sigma_{3}\right)d\varepsilon_{1}^{}+\frac{{\left(\sigma_{1}-\sigma_{3}\right)}^{2}}{2E}+\sigma_{3}\int d\varepsilon_{v}-\frac{\sigma_{3}\left(\text{1-2}\nu \right)\left(\sigma_{1}-\sigma_{3}\right)}{E}$$ (40)

Let the coefficients of the elastic stiffness matrix be represented as follows:

$$m_{1}=\frac{E_{f}(1-\nu_{f})}{\left(1+\nu_{f})(1-2\nu_{f}\right)}$$ (41)

It is evident that *m*_1_ changes continuously with the increase of axial strain. By substituting (22), (24), (25), (36), and (38) into (22), we can obtain the expression of the elastic-plastic stiffness matrix under conventional triaxial consolidated undrained conditions as follows:

$$\left[D^{ep}\right]=\left[\begin{array}{cc} m_{1}-\frac{n_{3}}{n_{9}} & \frac{2m_{1}\nu_{f}}{1-\nu_{f}}-\frac{n_{4}}{n_{9}}\\ \frac{m_{1}\cdot \nu_{f}}{1-\nu_{f}}-\frac{n_{5}}{n_{9}} & \frac{m_{1}}{1-\nu_{f}}-\frac{n_{6}}{n_{9}} \end{array}\right]$$ (42)

The expressions for the parameters in Eq (42) are as follows:

$$n_{1}=m_{1}(3I_{1}^{2}-K_{2}\sigma_{3}^{2}+\frac{2\nu_{f}}{1-\nu_{f}}(3I_{1}^{2}-K_{2}\sigma_{1}\sigma_{3}))$$ (43)

$$n_{2}=m_{1}(\frac{\nu_{f}}{1-\nu_{f}}(3I_{1}^{2}-K_{2}\sigma_{3}^{2})+\frac{1}{1-\nu_{f}}(3I_{1}^{2}-K_{2}\sigma_{1}\sigma_{3}))$$ (44)

$$n_{4}=\frac{m_{1}{}^{2}\times n_{1}}{I_{3}^{2}}\left[\frac{2\nu_{f}}{1-\nu_{f}}(3I_{1}^{2}I_{3}-\sigma_{3}^{2}I_{1}^{3})+\frac{1}{1-\nu_{f}}(3I_{1}^{2}I_{3}-\sigma_{1}\sigma_{3}I_{1}^{3}))\right]$$ (45)

$$n_{3}=\frac{m_{1}{}^{2}\times n_{1}}{I_{3}^{2}}\left[\left(3I_{1}^{2}I_{3}-\sigma_{3}^{2}I_{1}^{3})+\frac{\nu_{f}}{1-\nu_{f}}(3I_{1}^{2}I_{3}-\sigma_{1}\sigma_{3}I_{1}^{3})\right)\right]$$ (46)

$$n_{5}=\frac{m_{1}^{2}\times n_{2}}{I_{3}^{2}}\left[\left(3I_{1}^{2}I_{3}-\sigma_{3}^{2}I_{1}^{3})+\frac{\nu_{f}}{1-\nu_{f}}(3I_{1}^{2}I_{3}-\sigma_{1}\sigma_{3}I_{1}^{3})\right)\right]$$ (47)

$$n_{6}=\frac{m_{1}^{2}\times n_{2}}{I_{3}^{2}}\left[\frac{2\nu_{f}}{1-\nu_{f}}(3I_{1}^{2}I_{3}-\sigma_{3}^{2}I_{1}^{3})+\frac{1}{1-\nu_{f}}(3I_{1}^{2}I_{3}-\sigma_{1}\sigma_{3}I_{1}^{3}))\right]$$ (48)

$$n_{7}=\frac{m_{1}}{I_{3}^{2}}\left[\left(3I_{1}^{2}I_{3}-\sigma_{3}^{2}I_{1}^{3})+\frac{\nu_{f}}{1-\nu_{f}}(3I_{1}^{2}I_{3}-\sigma_{1}\sigma_{3}I_{1}^{3})\right)\right]$$ (49)

$$n_{8}=\frac{m_{1}^{}}{I_{3}^{2}}\left[\frac{2\nu_{f}}{1-\nu_{f}}(3I_{1}^{2}I_{3}-\sigma_{3}^{2}I_{1}^{3})+\frac{1}{1-\nu_{f}}(3I_{1}^{2}I_{3}-\sigma_{1}\sigma_{3}I_{1}^{3}))\right]$$ (50)

$$n_{9}=\frac{\left[1-\beta^{\prime }\left(f-f_{t}\right)\right]\sigma_{3}}{\alpha^{\prime }}\frac{m_{1}^{}}{I_{3}^{2}}\left[n_{7}(3I_{1}^{2}-K_{2}\sigma_{3}^{2})+n_{8}(3I_{1}^{2}-K_{2}\sigma_{1}\sigma_{3}))\right]$$ (51)

The values of *α’* and *β’* are determined through a combination of empirical observations and iterative calculations based on experimental data. With this, we have obtained the expression for the elastic-plastic matrix.

### 2.4 Determination of Structural Parameters

#### 2.4.1 Introduction to structural parameters

The evolution of volumetric strain damage ratio *λ* in soil is influenced by factors such as soil type, stress path, and stress history. In the early stages of loading, the structural integrity remains largely intact, resulting in a small value of *λ*. During this phase, the load is primarily borne by the elastic-brittle components; As the strain increases, the structural components in the soil gradually deteriorate, and the bonded components transform into friction components, leading to an increase in *λ*. When the specimen fails, it can be assumed that the majority of the structural components in the soil are destroyed, and *λ* approaches 1. Based on the trend of *λ*’s numerical changes, a functional expression for *λ* can be constructed.


$$\lambda =1-\text{exp}\left\{-\beta {\left(\alpha \varepsilon_{z}+\varepsilon_{x}+\varepsilon_{y}\right)}^{\psi }-{\left(\xi \varepsilon_{s}\right)}^{\theta }\right\}$$ (52)

The generalized shear strains $\varepsilon_{s}=\sqrt{2e_{ij}e_{ij}/3}$, *e*_*ij*_ = *ε*_*ij*_ − *ε*_*kk*_*δ*_*ij*_/3, and *δ*_*ij*_ are represented by Kronecker symbols. *α*, *β*, *ξ*, *ψ*, and *θ* are material parameters that are determined through continuous experimentation and adjustment. The z-axis represents the vertical direction, while the x-axis and y-axis are located in the horizontal plane. The expressions for the local strain coefficient matrix [*C*] and the local strain coefficient matrix *m*_3_ are constructed as follows:

$$\left[C\right]=\left[\begin{array}{cc} m_{3} & 0\\ 0 & m_{3} \end{array}\right]$$ (53)

$$m_{3}=\text{exp}\left(-{\left(t_{c}\times \varepsilon_{s}\right)}^{r_{c}}\right)$$ (54)

*ε*_s_ represents the generalized shear strain. *t*_*c*_ and *r*_*c*_ are material parameters that are determined through continuous experimentation and adjustment.

#### 2.4.2 Determination of structural parameters under triaxial stress conditions

Under triaxial stress conditions, Eq (52) can be expressed as:

$$\lambda =1-\text{exp}\left\{-\beta {\left(\alpha \varepsilon_{1}+2\varepsilon_{3}\right)}^{\psi }-{\left(\xi \frac{2}{3}\left(\varepsilon_{1}-\varepsilon_{3}\right)\right)}^{\theta }\right\}$$ (55)

Under triaxial stress conditions, the expression for the local strain coefficients *m*_3_ in Eq (54) is given by:

$$m_{3}=\text{exp}\left\{-{\left(t_{c}\frac{2}{3}\left(\varepsilon_{1}-\varepsilon_{3}\right)\right)}^{r_{c}}\right\}$$ (56)

The incremental expression for the local strain matrix $\left[C\right]=\left[\begin{array}{cc} m_{3} & 0\\ 0 & m_{3} \end{array}\right]$ in Eq (53) is given by:

$$\left[\Delta C\right]=\left[\begin{array}{cc} \frac{\partial m_{3}}{\partial \varepsilon_{1}}\Delta \varepsilon_{1}+\frac{\partial m_{3}}{\partial \varepsilon_{3}}\Delta \varepsilon_{3} & 0\\ 0 & \frac{\partial m_{3}}{\partial \varepsilon_{1}}\Delta \varepsilon_{1}+\frac{\partial m_{3}}{\partial \varepsilon_{3}}\Delta \varepsilon_{3} \end{array}\right]$$ (57)

$$\frac{\partial m_{3}}{\partial \varepsilon_{1}}\Delta \varepsilon_{1}+\frac{\partial m_{3}}{\partial \varepsilon_{3}}\Delta \varepsilon_{3}=\text{exp}\left\{-{\left[t_{c}\frac{2}{3}\left(\varepsilon_{{}_{1}}^{o}-\varepsilon_{3}^{o}\right)\right]}^{r_{c}}\right\}\times \frac{2}{3}\times r_{c}\times t_{c}\times {\left[t_{c}\frac{2}{3}\left(\varepsilon_{{}_{1}}^{o}-\varepsilon_{3}^{o}\right)\right]}^{r_{c}-1}\left(\Delta \varepsilon_{3}-\Delta \varepsilon_{1}\right)$$ (58)

$$m_{4}=\text{exp}\left\{-{\left[t_{c}\frac{2}{3}\left(\varepsilon_{{}_{1}}^{o}-\varepsilon_{3}^{o}\right)\right]}^{r_{c}}\right\}\times \frac{2}{3}\times r_{c}\times t_{c}\times {\left[t_{c}\frac{2}{3}\left(\varepsilon_{{}_{1}}^{o}-\varepsilon_{3}^{o}\right)\right]}^{r_{c}-1}$$ (59)

The Eq (57) can be rewritten as:

$$\left[\Delta C\right]=\left[\begin{array}{cc} m_{4}\left(\Delta \varepsilon_{3}-\Delta \varepsilon_{1}\right) & 0\\ 0 & m_{4}\left(\Delta \varepsilon_{3}-\Delta \varepsilon_{1}\right) \end{array}\right]$$ (60)

According to Eq (55), the incremental expression for the volumetric damage ratio *λ* is:

$$\Delta \lambda =\frac{\partial \lambda }{\partial \varepsilon_{1}}\Delta \varepsilon_{1}+\frac{\partial \lambda }{\partial \varepsilon_{3}}\Delta \varepsilon_{3}$$ (61)

Due to the smaller influence of $\frac{\partial \lambda }{\partial \varepsilon_{3}}\Delta \varepsilon_{3}$ compared to $\frac{\partial \lambda }{\partial \varepsilon_{1}}\Delta \varepsilon_{1}$ on *Δλ*, and considering the complexity of the expression for *λ*, for the purpose of simplification, only the influence of *Δε*_1_ on *Δλ* is considered. The expression is as follows:

$$\Delta \lambda \approx \frac{\partial \lambda }{\partial \varepsilon_{1}}\Delta \varepsilon_{1}=\text{exp}\left\{-\beta {\left(\alpha \varepsilon_{{}_{1}}^{o}+2\varepsilon_{{}_{3}}^{o}\right)}^{\psi }-{\left[\xi \frac{2}{3}(\varepsilon_{{}_{1}}^{o}-\varepsilon_{{}_{3}}^{o})\right]}^{\theta }\right\}\times \left\{\alpha \beta \psi {\left(\alpha \varepsilon_{{}_{1}}^{o}+2\varepsilon_{3}^{o}\right)}^{\psi -1}+\frac{2}{3}\xi \theta {\left[\xi \frac{2}{3}(\varepsilon_{{}_{1}}^{o}-\varepsilon_{{}_{3}}^{o})\right]}^{\theta -1}\right\}\times \Delta \varepsilon_{1}$$ (62)

### 2.5 Derivation of stress-strain relationship in triaxial stress state

We further derived the specific expression for stress increment in triaxial stress state. The coefficients of the stiffness matrix for brittle-elastic components are expressed as follows:

$$m_{2}=\frac{E_{vb}}{(1-\nu_{hhb})E_{vb}-2\nu_{vhb}^{2}E_{hb}}$$ (63)

According to Eqs. (41) and (63), after substitution, the stiffness matrix of the elastic-plastic components included in the Eq (21) and the stiffness matrix of the brittle-elastic components included in the Eq (18) can be respectively expressed as:

$$\left[D^{ep}\right]=\left[\begin{array}{cc} m_{1}-\frac{n_{3}}{n_{9}} & \frac{2m_{1}\nu_{f}}{1-\nu_{f}}-\frac{n_{4}}{n_{9}}\\ \frac{m_{1}\cdot \nu_{f}}{1-\nu_{f}}-\frac{n_{5}}{n_{9}} & \frac{m_{1}}{1-\nu_{f}}-\frac{n_{6}}{n_{9}} \end{array}\right]$$ (64)

$${\left[D\right]}_{b}=m_{2}\times \left[\begin{array}{cc} (1-\nu_{hhb})E_{vb} & 2\nu_{vhb}E_{hb}\\ \nu_{vhb}E_{hb} & E_{hb} \end{array}\right]=\left[\begin{array}{cc} m_{2}\times (1-\nu_{hhb})E_{vb} & 2m_{2}\times \nu_{vhb}E_{hb}\\ m_{2}\times \nu_{vhb}E_{hb} & m_{2}\times E_{hb} \end{array}\right]$$ (65)

Expanding the stress increment expression for general stress state given by the Eq (16) we obtain the following expression:

$$\left\{\begin{array}{c} \Delta \sigma_{1}\\ \Delta \sigma_{3} \end{array}\right\}=\left\{\begin{array}{c} \begin{array}{l} n_{11}\Delta \varepsilon_{1}+n_{12}\Delta \varepsilon_{3}+n_{13}\varepsilon_{1}{}^{o}(\Delta \varepsilon_{3}-\Delta \varepsilon_{1})+n_{14}\varepsilon_{3}{}^{o}(\Delta \varepsilon_{3}-\Delta \varepsilon_{1})\\ +n_{15}\varepsilon_{1}{}^{o}+n_{16}\varepsilon_{3}{}^{o}+\frac{\Delta \lambda }{\lambda^{o}}(\sigma_{1}{}^{o}-\sigma_{1}{{}_{b}}^{o}) \end{array}\\ \begin{array}{l} n_{21}\Delta \varepsilon_{1}+n_{22}\Delta \varepsilon_{3}+n_{23}\varepsilon_{1}{}^{o}(\Delta \varepsilon_{3}-\Delta \varepsilon_{1})+n_{24}\varepsilon_{3}{}^{o}(\Delta \varepsilon_{3}-\Delta \varepsilon_{1})\\ +n_{25}\varepsilon_{1}{}^{o}+n_{26}\varepsilon_{3}{}^{o}+\frac{\Delta \lambda }{\lambda^{o}}(\sigma_{3}{}^{o}-\sigma_{3}{{}_{b}}^{o}) \end{array} \end{array}\right\}$$ (66)

In the Eq (66), the expressions for some parameter symbols are as follows:

$$n_{11}=\left\{\left(m_{1}-\frac{n_{3}}{n_{9}}\right)+(1-\lambda^{o})\left[m_{2}\times m_{3}\times (1-\nu_{hhb})\times E_{vb}-\left(m_{1}-\frac{n_{3}}{n_{9}}\right)\times m_{3}\right]\right\}$$ (67)

$$n_{12}=\left\{\left(\frac{2m_{1}\times \nu_{f}^{o}}{1-\nu_{f}^{o}}-\frac{n_{4}}{n_{9}}\right)+(1-\lambda^{o})\left[2m_{2}\times m_{3}\times \nu_{v}{}_{hb}\times E_{hb}-\left(\frac{2m_{1}\times \nu_{f}^{o}}{1-\nu_{f}^{o}}-\frac{n_{4}}{n_{9}}\right)\times m_{3}\right]\right\}$$ (68)

$$n_{13}=\left\{(1-\lambda^{o})\left[m_{2}\times m_{4}\times (1-\nu_{hhb})\times E_{vb}-\left(m_{1}-\frac{n_{3}}{n_{9}}\right)\times m_{4}\right]\right\}$$ (69)

$$n_{14}=\left\{(1-\lambda^{o})\left[2m_{2}\times m_{4}\times \nu_{v}{}_{hb}\times E_{hb}-\left(\frac{2m_{1}\times \nu_{f}^{o}}{1-\nu_{f}^{o}}-\frac{n_{4}}{n_{9}}\right)\times m_{4}\right]\right\}$$ (70)

$$n_{15}=-\left\{\frac{\Delta \lambda \times \left(m_{1}-\frac{n_{3}}{n_{9}}\right)\times (1-m_{3})}{\lambda^{o}}\right\}$$ (71)

$$n_{16}=-\left\{\frac{\Delta \lambda }{\lambda^{o}}\times \left(\frac{2m_{1}\times \nu_{f}^{o}}{\left(1-\nu_{f}^{o}\right)}-\frac{n_{4}}{n_{9}}\right)\times (1-m_{3})\right\}$$ (72)

$$n_{21}=\left\{\left(\frac{m_{1}\times \nu_{f}^{o}}{1-\nu_{f}^{o}}-\frac{n_{5}}{n_{9}}\right)+(1-\lambda^{o})\left[m_{2}\times m_{3}\times \nu_{v}{}_{hb}\times E_{hb}-\left(\frac{m_{1}\times \nu_{f}^{o}}{1-\nu_{f}^{o}}-\frac{n_{5}}{n_{9}}\right)\times m_{3}\right]\right\}$$ (73)

$$n_{22}=\left\{\left(\frac{m_{1}}{1-\nu_{f}^{o}}-\frac{n_{6}}{n_{9}}\right)+(1-\lambda^{o})\left[m_{2}\times m_{3}\times E_{hb}-\left(\frac{m_{1}}{1-\nu_{f}^{o}}-\frac{n_{6}}{n_{9}}\right)\times m_{3}\right]\right\}$$ (74)

$$n_{23}=\left\{(1-\lambda^{o})\left[m_{2}\times m_{4}\times \nu_{v}{}_{hb}\times E_{hb}-\left(\frac{m_{1}\times \nu_{f}^{o}}{1-\nu_{f}^{o}}-\frac{n_{5}}{n_{9}}\right)\times m_{4}\right]\right\}$$ (75)

$$n_{24}=\left\{(1-\lambda^{o})\left[m_{2}\times m_{4}\times E_{hb}-\left(\frac{m_{1}}{1-\nu_{f}^{o}}-\frac{n_{6}}{n_{9}}\right)\times m_{4}\right]\right\}$$ (76)

$$n_{25}=-\left\{\frac{\Delta \lambda }{\lambda^{o}}\times \left(\frac{m_{1}\times \nu_{f}^{o}}{1-\nu_{f}^{o}}-\frac{n_{5}}{n_{9}}\right)\times (1-m_{3})\right\}$$ (77)

$$n_{26}=-\left\{\frac{\Delta \lambda }{\lambda^{o}}\times \left(\frac{m_{1}}{1-\nu_{f}^{o}}-\frac{n_{6}}{n_{9}}\right)\times (1-m_{3})\right\}$$ (78)

The expression for the stress increment corresponding to the initial stress state, as expanded from Eq (17), is given as follows:

$$\left\{\begin{array}{c} \Delta \sigma_{1}\\ \Delta \sigma_{3} \end{array}\right\}=\left\{\begin{array}{c} n_{11}\Delta \varepsilon_{1}+n_{12}\Delta \varepsilon_{3}+n_{13}\varepsilon_{1}{}^{o}(\Delta \varepsilon_{3}-\Delta \varepsilon_{1})+n_{14}\varepsilon_{3}{}^{o}(\Delta \varepsilon_{3}-\Delta \varepsilon_{1})\\ n_{21}\Delta \varepsilon_{1}+n_{22}\Delta \varepsilon_{3}+n_{23}\varepsilon_{1}{}^{o}(\Delta \varepsilon_{3}-\Delta \varepsilon_{1})+n_{24}\varepsilon_{3}{}^{o}(\Delta \varepsilon_{3}-\Delta \varepsilon_{1}) \end{array}\right\}$$ (79)

In the undrained consolidation test, *Δσ*_1_ and *Δσ*_3_ represent the increments of axial and radial effective stresses, respectively. Initially, the pore water pressure in the specimen is zero, and the radial effective stress *σ*_3_ is equal to the confining pressure. During the test, the radial total stress remains constant, and the algebraic sum of *Δσ*_3_ and the increment of the pore water pressure is zero, that is:

$$\Delta p=-\Delta \sigma_{3}$$ (80)

By continuously experimenting and adjusting, we can determine the appropriate material parameters, which allow us to solve for the variations of the deviatoric stress and pore water pressure with axial strain.

## 3 Triaxial compression tests

This study adopted the preparation method proposed by Liu and Luo [27] and Luo et al. [28]. The dried powdered clay, kaolin, 32.5R cement and NaCl particles were sieved through a 0.5 mm sieve, and mixed in mass ratios of 65%, 20%, 5% and 10%, and then compacted in a φ 5 cm×10 cm three-valve membrane at a dry density of 1.49 g/cm^3^, and saturated using the evacuation method. The specimens were placed in flowing water with a flow rate of 8 cm^3^/s for 7 d to produce isotropic structural soil are shown in Table 3. The initial stress isotropic structural soil with transverse isotropy was prepared by applying 100 kPa pre-stress at the end of the specimen during the conditioning in flowing water, and the sample was compressed in a side-limiting compression state due to the rigid constraints of the triaxial membrane, and the initial stress anisotropic structural soil with transverse isotropy was prepared after the conditioning for 7 d in the flowing water.

**Table 3:** Proportioning of structured soil sample.

Classification	Silt clay	Kaolin clay	Cement	Salt
Mass percentage	65%	20%	5%	10%

After the salt particles in the structured soil are washed away by flowing water, the dry density of the structural soil will change from 1.49 g/cm^3^ to 1.34 g/cm^3^. After the completion of the experiment, the structured soil samples are dried, crushed, and sieved through a 0.5mm sieve. Subsequently, the soil material is compacted in a triaxial membrane with a diameter of Φ5.00cm and a height of 10cm at a density of 1.34 g/cm^3^, in 4 layers. The samples are saturated using a vacuum saturation method and then placed in a water tank for further use. It is worth noting that during the process of drying, crushing, and sieving the experimentally prepared structural soil samples, the structure of the samples will be completely destroyed. In other words, the prepared remolded soil samples will not possess structural characteristics.

### 3.1 Experimental results of consolidated undrained triaxial compression test on structured soils

The initial stress anisotropic structured soil, isotropic structured soil, and remolded soil were subjected to consolidated undrained (CU) triaxial compression tests under the consolidation stresses of 25kPa, 50kPa, 100kPa and 200kPa respectively.

#### 3.1.1 Experimental results for initial stress anisotropic structured soil

**Fig 1:**
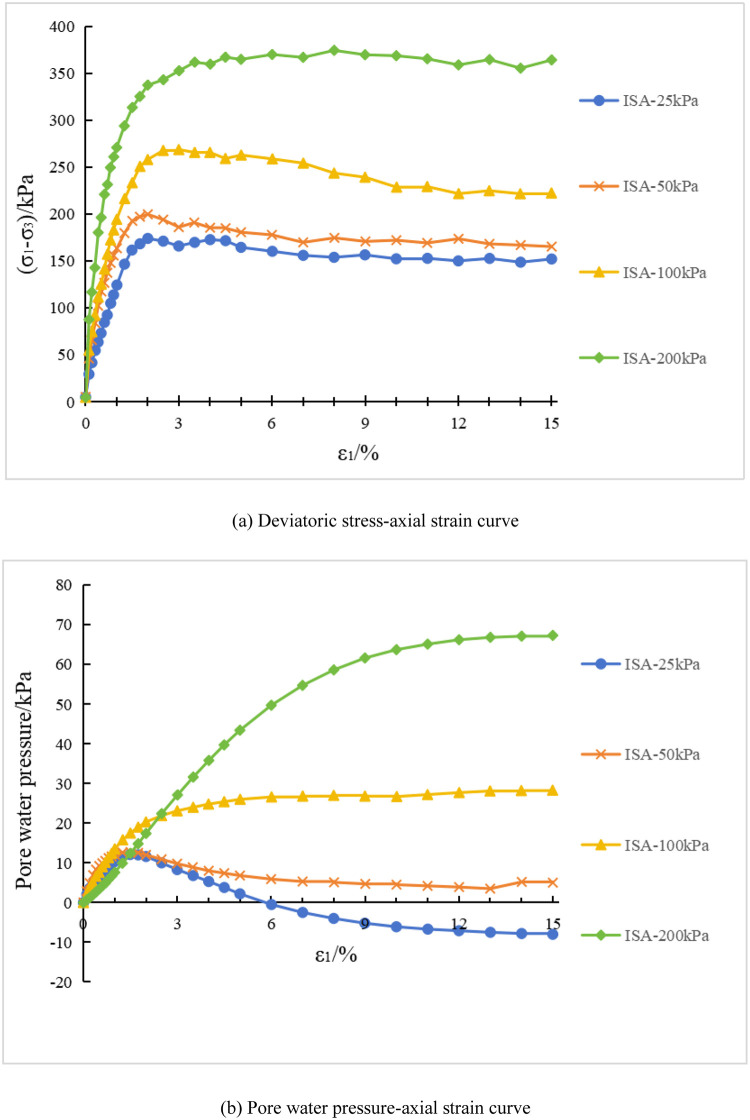
Test results of initial stress anisotropic structured soil(ISA). (a) Deviatoric stress-axial strain curve. (b) Pore water pressure-axial strain curve.

**Fig 2:**
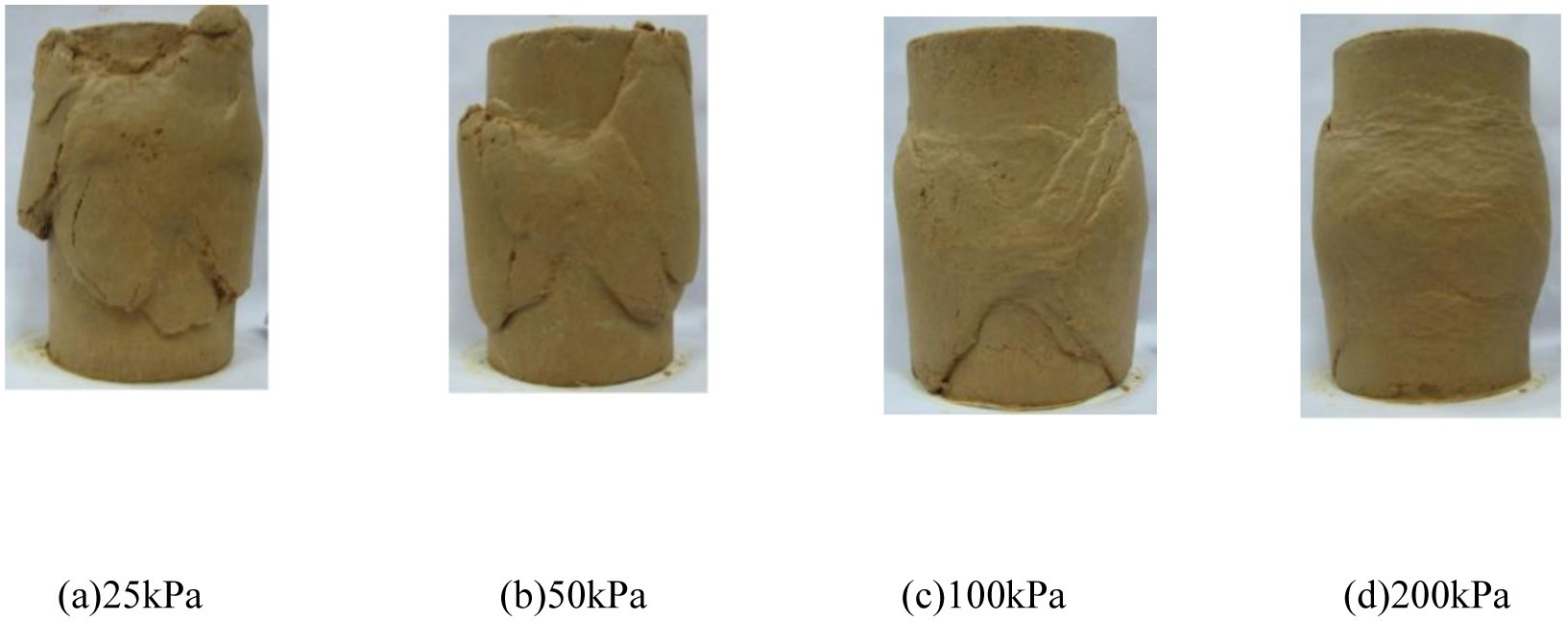
Failure pattern of initial stress anisotropic structured soil (ISA). (a) 25kPa. (b) 50kPa. (c) 100kPa. (d) 200kPa.

Fig 1(a) and 1(b) show the deviatoric stress and pore water pressure-strain curves, respectively, for the conventional triaxial compression tests conducted on the initial stress anisotropic structured soil. In Fig 1(a) and 1(b), "ISA-25" represents the test curve of the initial stress anisotropic structured soil (ISA) specimen under a consolidation stress of 25 kPa, and so on for the remaining curves. Fig 2(a)–2(d) show the failure modes of each initial stress anisotropic structured soil (ISA) specimen. In Fig 1(a) and 1(b), the deviatoric stress and pore water pressure of the specimens both increase with the increase of consolidation stress. At lower consolidation stresses, during the experimental process, the deviatoric stress increases to a certain value and then gradually decreases. The deviatoric stress-axial strain curve exhibits a peak, indicating a significant strain softening. The pore water pressure-axial strain curve of the specimen also exhibits a peak at this stage. When the specimen is subjected to loading, it initially shows an increase in pore water pressure (corresponding to the shear contraction phenomenon under consolidated-drained conditions), and then gradually decreases after reaching the peak pore water pressure (corresponding to the dilatancy trend under consolidated-drained conditions). Specifically, at a confining pressure of 25 kPa, the specimen exhibits a negative pore water pressure during the later stages of the test, indicating that the effective stress in the specimen is higher than the fully consolidated state. When the confining pressure is 100 kPa, the deviatoric stress-axial strain curve of the specimen still exhibits a peak, but with a certain degree of attenuation. The pore water pressure reaches a certain value and then remains relatively stable. The pore water pressure-axial strain curve does not show a clear peak, indicating the absence of dilatancy trend in the specimen. Due to the influence of positive pore water pressure, the lateral effective stress of the specimen continues to decrease during the shearing process; At confining pressures of 200kPa, the deviatoric stress and pore water pressure of the specimen continue to increase with the increase in axial strain, exhibiting strain hardening characteristics. This corresponds to the sustained shear compaction observed in conventional triaxial compression tests under consolidated drained conditions. Correspondingly, in Fig 2, for lower confining pressures, the specimen exhibits a clear shear band during failure. For higher confining pressures, the specimen shows overall bulging failure with localized irregularities.

#### 3.1.2 Experimental results of isotropic structured soil

**Fig 3:**
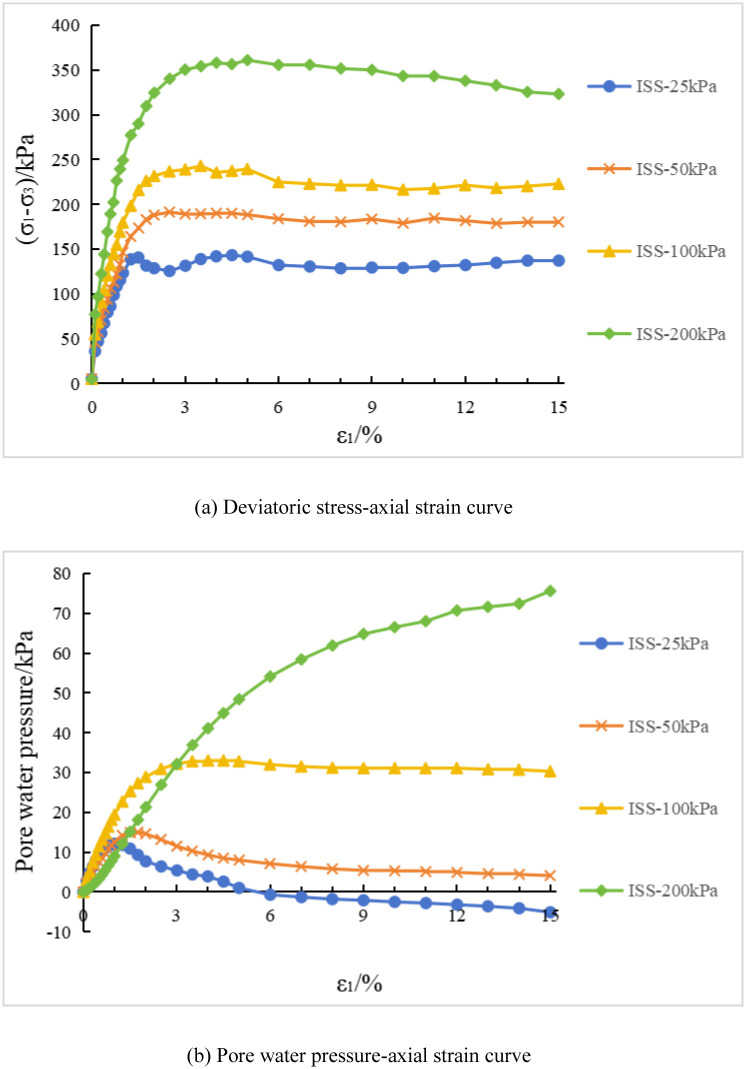
Test results of isotropic structural soil(ISS). (a) Deviatoric stress-axial strain curve. (b) Pore water pressure-axial strain curve.

**Fig 4:**
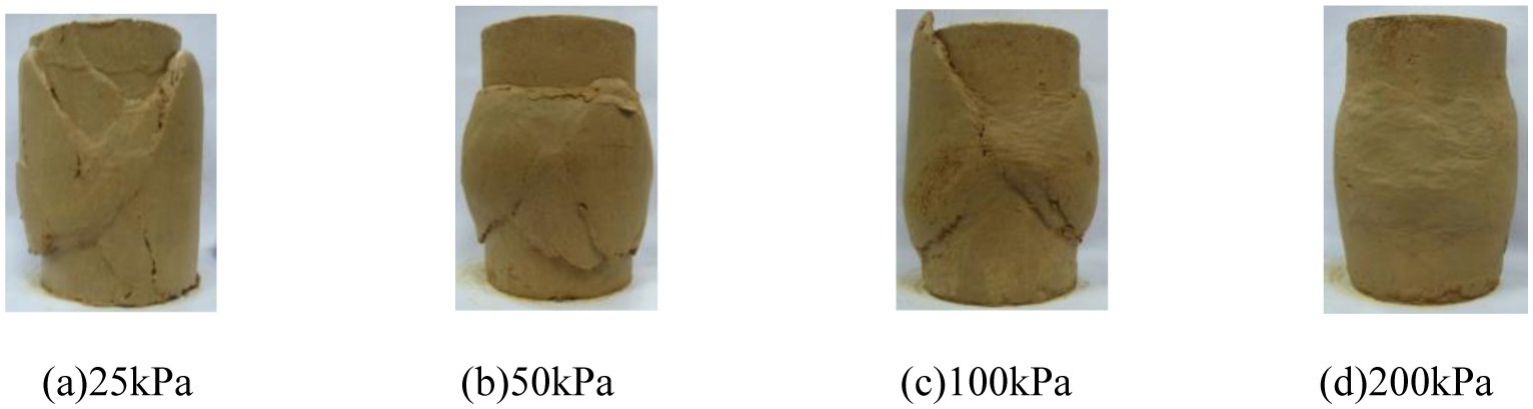
Failure pattern of initially stressed structurally isotropic soil(ISS). (a) 25kPa. (b) 50kPa. (c) 100kPa. (d) 200kPa.

The deviatoric stress-axial strain curve and pore water pressure-axial strain curve of isotropic structured soil under conventional triaxial compression test are shown in Fig 3(a) and 3(b). In Fig 3(a) and 3(b), "ISS-25" represents the test curve of the initial stress isotropic structured soil(ISS) specimen under a consolidation stress of 25 kPa, and so on for the remaining curves. Fig 4(a) and 4(b) show the failure patterns of each specimen.

In Fig 3, the deviatoric stress and pore water pressure of the specimen increase with the increase of confining stress. This observation is similar to the experimental results of initial stress anisotropic structured soil. At lower consolidation stresses, the specimen exhibits a peak in the deviatoric stress-axial strain curve. Similarly, the specimen shows a noticeable strain softening behavior, and the corresponding pore water pressure-axial strain curve exhibits a peak. At a confining pressure of 25 kPa, the specimen exhibits negative pore water pressure during the later stages of the test, corresponding to the phenomenon of volumetric contraction followed by dilatancy observed in consolidated-drained triaxial test. At confining pressures of 200 kPa, the specimen exhibits strain hardening behavior, and the pore water pressure continues to increase. This corresponds to the phenomenon of sustained shear contraction observed in consolidated-drained triaxial test. Correspondingly, in Fig 4, at lower confining pressure, the specimen exhibits distinct shear bands during failure. At higher confining pressure, the specimen displays a bulging failure pattern, similar to the case of initial stress anisotropic structured soil.

#### 3.1.3 Experimental results of remolded soil

**Fig 5:**
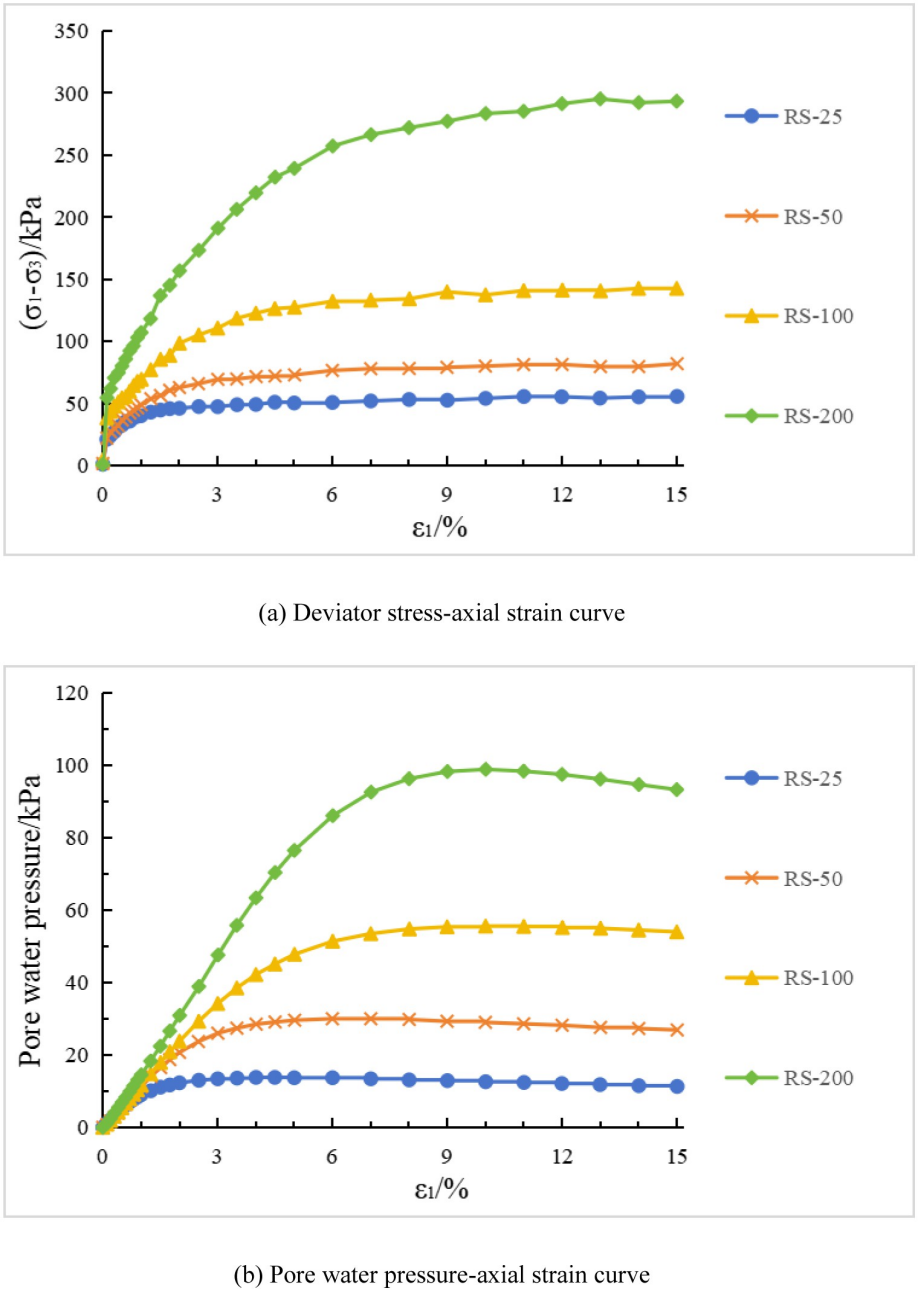
Remolded soil test curve(RS). (a) Deviator stress-axial strain curve. (b) Pore water pressure-axial strain curve.

**Fig 6:**
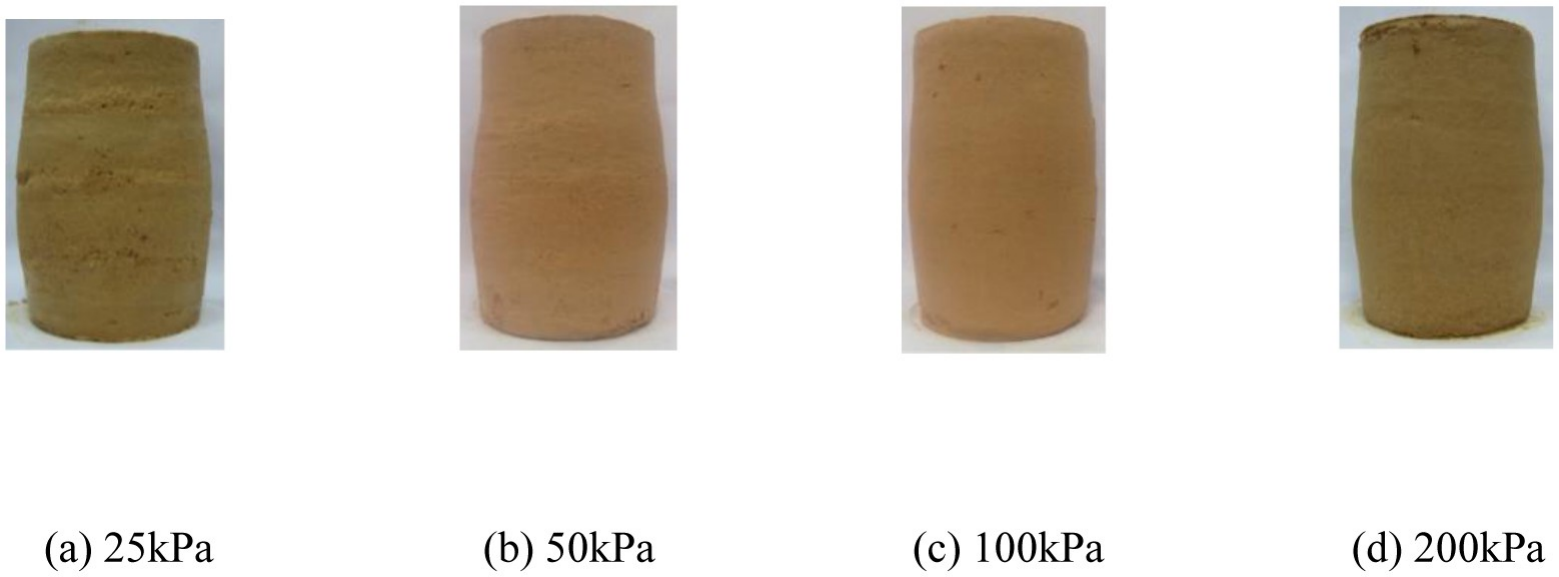
Failure pattern of remolded soil(RS). (a) 25kPa. (b) 50kPa. (c) 100kPa. (d) 200kPa.

The deviatoric stress-strain curves and pore water pressure-strain curves of the remolded soil under conventional triaxial compression test are shown in Fig 5(a) and 5(b). In Fig 5(a) and 5(b), "RS-25" represents the test curve of the remolded soil (RS) specimen under a consolidation stress of 25 kPa, and so on for the remaining curves. Fig 6(a)–6(e) show the failure patterns of each specimen. The deviatoric stress and pore water pressure curves of the remolded soil specimens increase with increasing confining pressure. Unlike structured soil, in the case of remolded soil specimens tested at the five different confining pressures, the deviatoric stress tends to stabilize after reaching a certain value. Additionally, the deviatoric stress curve does not exhibit significant peaks, indicating a strain hardening behavior of the specimens. In Fig 6, unlike structured soil, the remolded soil specimens do not exhibit structural features. Therefore, there is no presence of shear bands during failure. Although there may be slight unevenness on the surface of the specimens at low confining pressures, the overall failure pattern is characterized by bulging. The failure pattern of structured soil under high consolidation stresses is similar to the failure pattern observed in remolded soil.

### 3.2 Experimental results analysis

**Fig 7:**
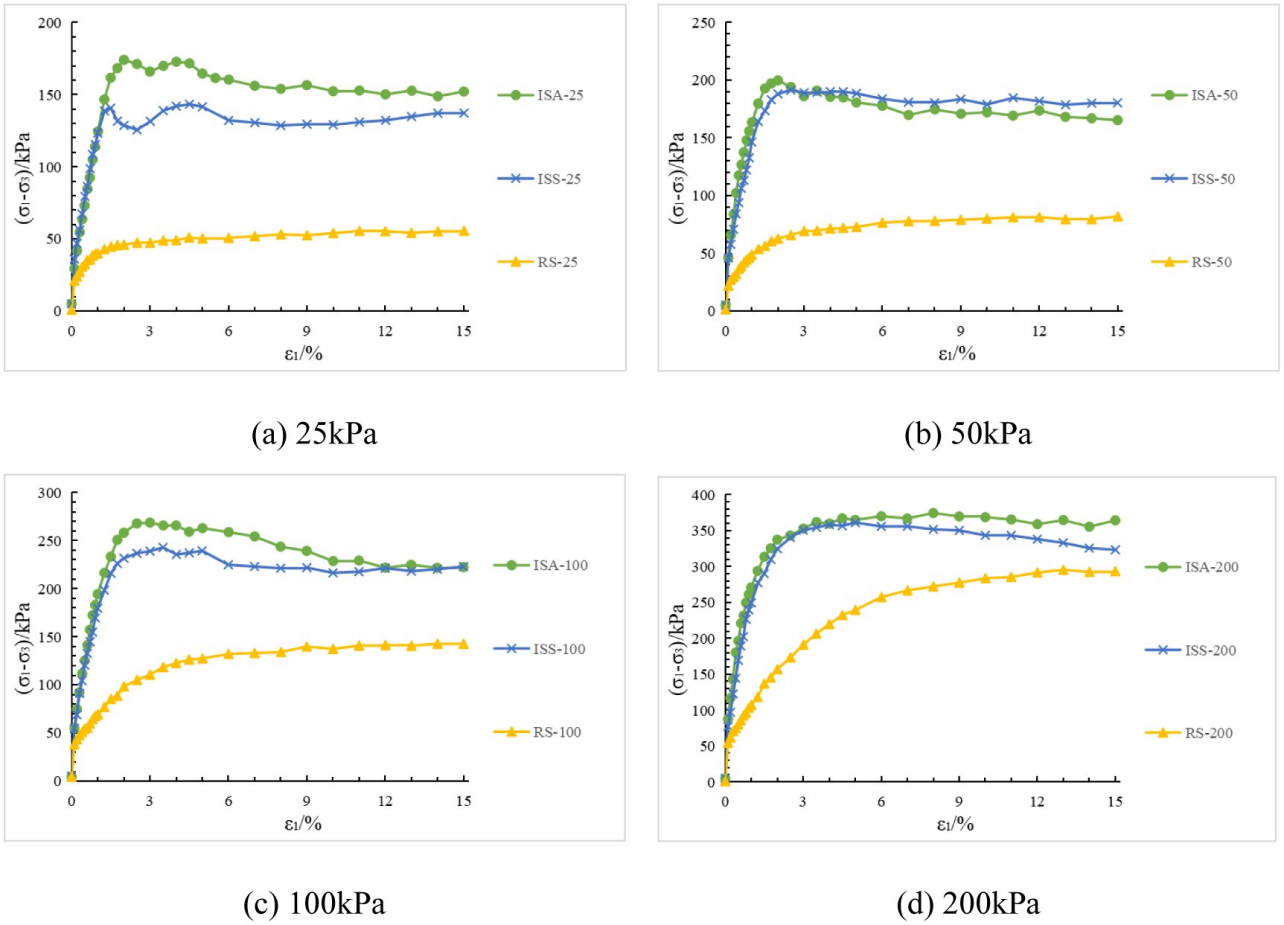
Deviatoric stress-strain curves for the three soil types under the same confining pressure. (a) 25kPa. (b) 50kPa. (c) 100kPa. (d) 200kPa.

Under the same confining pressure conditions, the deviatoric stress-strain and pore water pressure-strain curves of structured soil with initial stress anisotropy, structured soil with isotropic structure, and remolded soil are shown in Fig 7(a) and 7(b).

**Fig 8:**
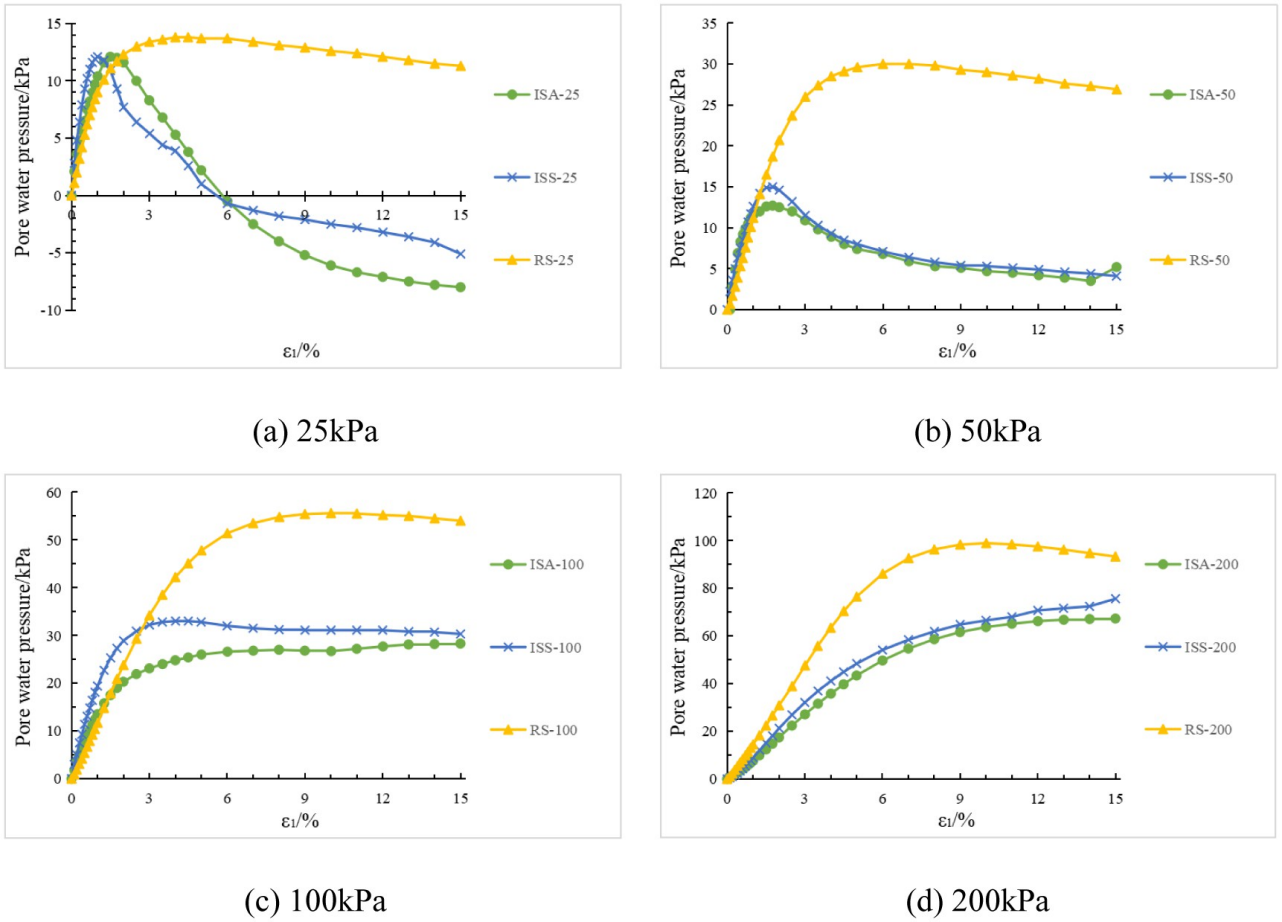
Pore water pressure-strain curves for the three soil types under the same confining pressure. (a) 25kPa. (b) 50kPa. (c) 100kPa. (d) 200kPa.

Based on Figs 7 and 8, an analysis is conducted on the results of triaxial compression tests for the two types of structured soil and remolded soil under consolidated undrained conditions. Under the four different confining pressures, the remolded soil exhibits strain hardening behavior, with the pore pressure values showing an overall increasing trend. This corresponds to the phenomenon of sustained shear contraction observed in consolidated-drained tests. At lower confining pressures (25 kPa and 50 kPa), both the initial stress anisotropic structured soil and the isotropic structured soil exhibit distinct yield strengths. Their deviatoric stress-strain curves show clear peaks, indicating strain softening behavior. As the axial strain increases, the deviatoric stress (σ_1_-σ_3_) of the structured soil remains relatively stable; at a confining pressure of 100 kPa, both types of structured soil approach plastic flow behavior; At higher confining pressures (200 kPa), the deviatoric stresses of the various types of structural soils continued to increase over the course of the tests, suggesting the presence of strain-hardening behaviour. For ISA, the deviatoric stress (σ_1_-σ_3_) tends to stabilise at around 375 kPa to show strain hardening; for ISS, the deviatoric stress (σ_1_-σ_3_) decreases after it reaches its peak (370 kPa), showing strain softening. In addition, the curves for the structural soils tended to approach the deviatoric stress curves for the remoulded soils in the later stages of the tests. Correspondingly, under low confining pressures, the pore water pressure-axial strain curves of the structured soils exhibit distinct peaks. At a confining pressure of 25 kPa, negative pore water pressure may even occur. At this stage, the soil skeleton bears a greater load compared to after consolidation, which corresponds to the phenomenon observed in consolidated-drained tests, where volumetric contraction is followed by dilation; at a confining pressure of 100 kPa, the pore water pressure of both structured soils reaches a certain value and then stabilizes, indicating a state of volume stability under consolidated-drained conditions. Under higher confining pressures, the pore water pressure of the structured soils continues to increase and then slightly decreases, following a similar pattern as observed in remolded soil. Overall, as the confining pressure increases, the mechanical characteristics of the structured soils gradually approach those of the remolded soil. During the testing process, the maximum deviatoric stress of the structured soils is greater than that of the remolded soil. Among them, the structurally stronger initial stress anisotropic structured soil exhibits higher deviator stress compared to the isotropic structured soil. However, the variation pattern of pore water pressure is opposite to that of deviatoric stress for all three soil types.

The above phenomena is analyzed using the theory of the Binary-medium model (the microscopic units can indeed be divided into elastic-brittle and elastic-plastic elements). Remolded soil is prepared from crushed, dried, and sieved specimens of structured soil. The remolded specimens do not possess any bonding among soil grains, and the external load is solely borne by the friction components (also known as elasto-plastic components); in structured soil, there are not only friction components but also bonded components (also known as brittle-elastic components) that exhibit brittle-elastic behavior. The external load on the structured soil is shared by both the parallel combination of brittle-elastic components and elasto-plastic components until the localized load on the brittle-elastic components exceeds their yield strength. At this point, the brittle-elastic components will fracture and transform into elasto-plastic components. During the curing process of structured soil, the initial stress anisotropic structured soil is influenced by the axial load and radial rigid constraints. The strength of the bonded components inside the specimen is greater than that of the isotropic structured soil. Considering the compression of the specimen under the initial consolidation stress, the void ratio is smaller than that of the isotropic structured soil. Therefore, the strength of its friction components is also stronger than that of isotropic structured soil.

At a confining pressure of 25 kPa, during the initial stages of the test, the external load in the structured soil is primarily borne by the bonded components, with their strength exceeding that of the friction components. As a result, the deviatoric stress in the structured soil is significantly greater than that in the remolded soil, with the initial stress anisotropic structured soil exhibiting higher deviatoric stress than the isotropic structured soil; Correspondingly, in the structured soil, the soil skeleton bears a greater load, resulting in lower pore water pressures compared to the remolded soil. Specifically, the initial stress anisotropic structured soil exhibits lower pore water pressures than the isotropic structured soil. The pore water pressures in all three soil types continue to increase, corresponding to the shearing and contraction phenomenon under the conditions of consolidation and drainage. As the axial strain increases, the bonded components undergo fragmentation and transform into frictional components. Under low confining pressure conditions, the increase in strength of the frictional components is not sufficient to compensate for the reduction in strength of the bonded components. Therefore, the shear strength of the structured soil weakens, resulting in strain softening. In the later stages of the test, it is assumed that most of the bonded components in the structured soil undergoes damage. It is further assumed that the rate of damage to the bonded components is the same in both the structured soil with initial anisotropic stress and the structured soil with isotropic stress. However, the bonded components in the structured soil with initial anisotropic stress are capable of bearing a higher load. Considering only the frictional components in the structured soil, the initial anisotropic structured soil has a lower void ratio than the isotropic structured soil. The initial anisotropic structured soil exhibits a more pronounced tendency for dilation compared to the isotropic structured soil. This is similar to the case where dense sand is more prone to shear dilation compared to loose sand. The presence of distinct shear bands during the failure of structured soil specimens is an important factor contributing to strain softening in structured soils.

**Fig 9:**
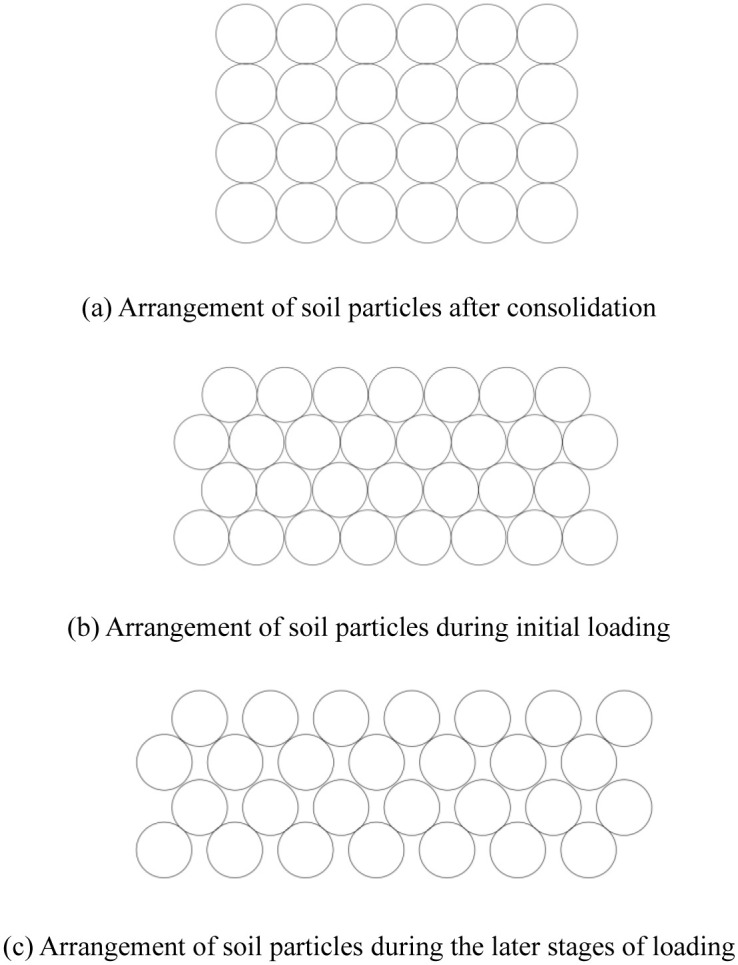
Illustration of changes in the arrangement of soil particles during the loading process of remolded soil. (a) Arrangement of soil particles after consolidation. (b) Arrangement of soil particles during initial loading. (c) Arrangement of soil particles during the later stages of loading.

As the confining pressure increases, the structured soil experiences an increase in the damage rate of its bonded components during the consolidation process. The presence of shear bands gradually diminishes during specimen failure. Consequently, the strain softening and shear dilation tendencies of the structured soil decrease. Overall, the maximum deviatoric stress and maximum pore water pressure values correspond to increasing axial strains. Specifically, at higher confining pressures, the pore water pressure in remolded soil reaches a peak and then slightly decreases. There seems to be a tendency for shear dilation, which is different from the shear dilation observed in structured soil at lower confining pressures. The possible reason for this is that at higher confining pressures, remolded soil, which lacks bonding among soil grains, has closely packed soil particles after consolidation. Therefore, it can be assumed that the arrangement of soil particles at this stage is as shown in Fig 9(a); Under the initial application of load, the soil particles in remolded soil further compact, gradually transitioning to the condition depicted in Fig 9(b). Macroscopically, this corresponds to volume reduction under consolidation drainage conditions and an increase in pore water pressure under consolidated undrained conditions; As the load continues to increase, the soil particles transition from loose state to a dense state until reaching the state depicted in Fig 9(c). At this point, the inter-particle voids increase compared to Fig 9(b), exhibiting a shear dilation trend. Macroscopically, this is manifested as a slight decrease in pore water pressure under undrained conditions of consolidation.

## 4 Determination of model parameter and model verification

After repeated adjustments, the material parameters of the binary-medium constitutive model have been determined. The parameters corresponding to the initial stress anisotropic structured soil and isotropic structured soil are presented in Table 4, while the material parameters for isotropic structured soil are listed in Table 5.

**Table 4:** Material parameters of initial stress anisotropic structured soil.

Confining pressure	25kPa	50kPa	100kPa	200kPa
*α*	110.0	60.0	40.0	34.0
*β*	1.4	1.63	1.85	2.10
*ξ*	8	19.0	32.0	33.0
*ψ*	0.7	0.82	1.0	1.2
*θ*	0.9	1.0	1.15	1.32
*t*_*c*_	1.0	1.0	1.0	10
*r*_*c*_	2.0	1.95	1.9	1.8
*α′*	-24	-52	-105	-210
*β′*	0.01	0.01	0.01	0.01

**Table 5:** Material parameters of isotropic structured soil.

Confining pressure	25kPa	50kPa	100kPa	200kPa
*α*	160.0	70.0	40.0	34.0
*β*	1.4	1.63	1.85	2.10
*ξ*	8	19.0	32.0	33.0
*ψ*	0.7	0.82	1.0	1.2
*θ*	0.9	1.0	1.15	1.32
*t*_*c*_	1.0	1.0	1.0	10
*r*_*c*_	2.0	1.95	1.9	1.8
*α′*	-24	-52	-105	-210
*β′*	0.01	0.01	0.01	0.01

**Fig 10:**
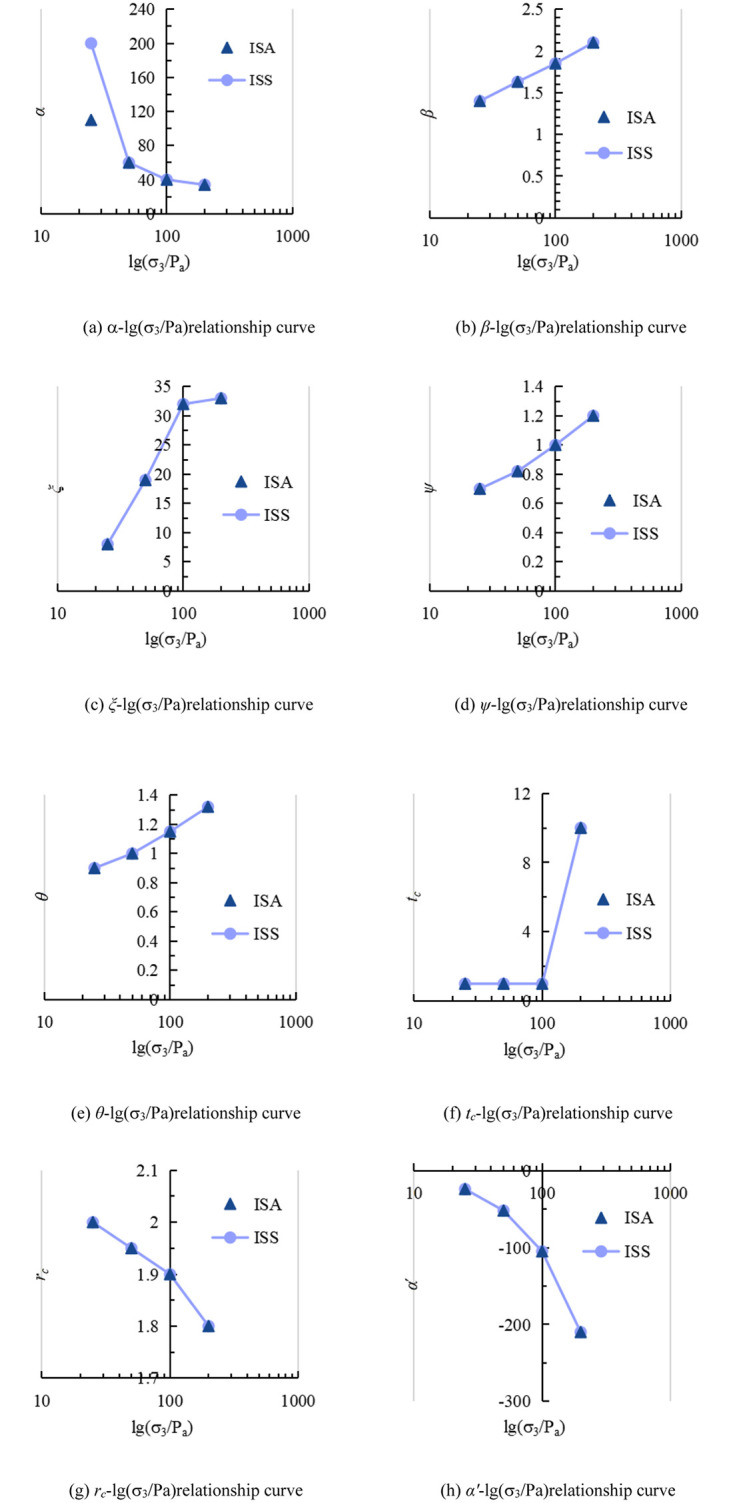
Variation pattern of material parameters for the two structured soils. (a) α-lg(σ_3_/Pa)relationship curve. (b) *β*-lg(σ_3_/Pa)relationship curve. (c) *ξ*-lg(σ_3_/Pa)relationship curve. (d) *ψ*-lg(σ_3_/Pa)relationship curve. (e) *θ*-lg(σ_3_/Pa)relationship curve. (f) *t*_*c*_-lg(σ_3_/Pa)relationship curve. (g) *r*_*c*_-lg(σ_3_/Pa)relationship curve. (h) *αʹ*-lg(σ_3_/Pa)relationship curve.

The variation of material parameters with axial strain in these two types of structured soil can be seen in Fig 10(a)–10(h), where ISA represents the initial stress anisotropic structured soil, and ISS represents the isotropic structured soil.

According to Tables 4 and 5 and Fig 10(a)–10(h), it can be observed that *β’* remains relatively constant and does not fluctuate significantly with structural variations in the constitutive model. Due to the preloading effect during the curing process, the initial stress anisotropic structured soil exhibits stronger structural properties compared to the isotropic structured soil. As the confining pressure increases, the structural characteristics in the soil gradually weaken; As the confining pressure increases, *α* gradually decreases in both types of structured soils. On the other hand, *β*, *θ*, *ψ*, and *ξ* increase with the increase in confining pressure, indicating that these parameters tend to increase as the structural properties weaken. Overall, the trend of *t*_c_ is similar to that of *ξ*. Both *r*_c_ and *α*ʹ decrease with the increase in confining pressure, and under the same confining pressure conditions, these material parameters are the same for both types of structured soils.

Due to different curing methods, there are differences in the structural properties of the two types of structured soils. However, in this case, the structural differences between the two types of structured soils are relatively small compared to the structural differences that occur with the increase in confining pressure in similar samples. Therefore, in Fig 10(a)–10(h), the material parameters of the two types of structured soils show only slight differences or no differences.

**Fig 11:**
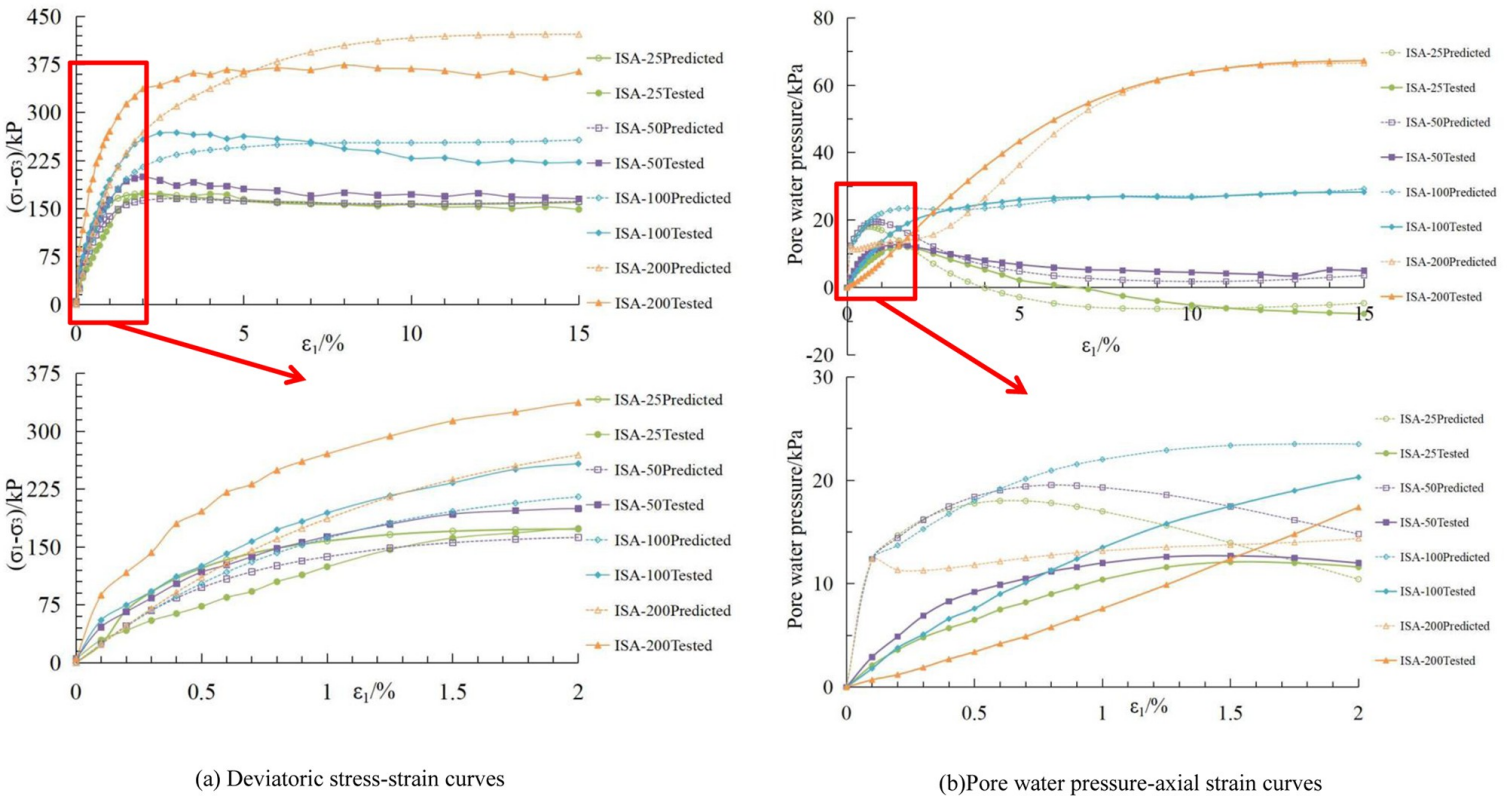
Comparison between calculated and experimental curves for initial stress anisotropic structured soil. (a) Deviatoric stress-strain curves. (b) Pore water pressure-axial strain curves.

**Fig 12:**
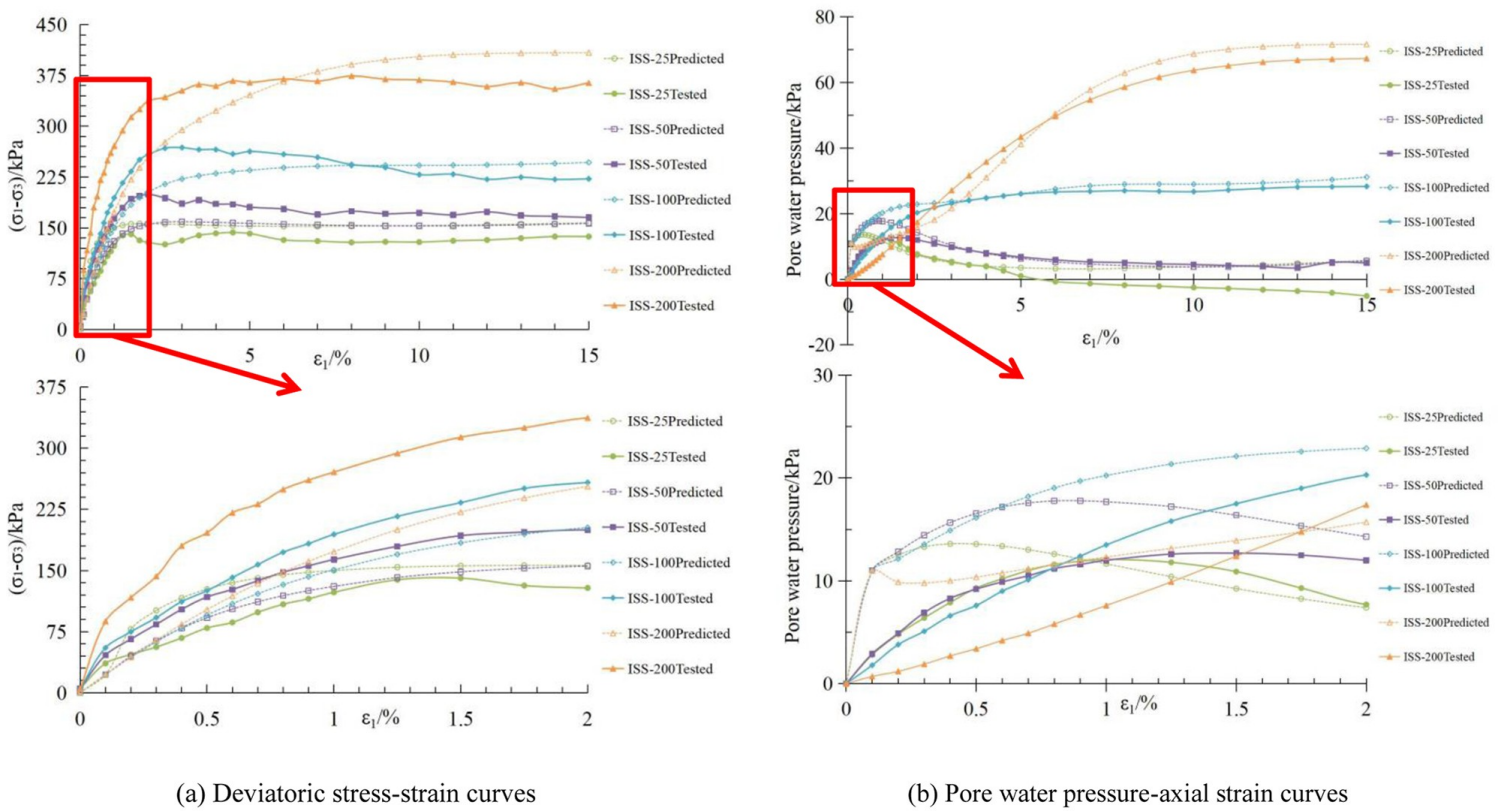
Comparison between calculated and experimental curves for isotropic structured soil. (a) Deviatoric stress-strain curves. (b) Pore water pressure-axial strain curves.

The stress-deviator strain and pore water pressure-axial strain curves for the initial stress anisotropic structured soil and isotropic structured soil, obtained using the aforementioned material parameters, are shown in Figs 11(a), 11(b), 12(a) and 12(b), respectively.

Based on Figs 11(a), 11(b), 12(a) and 12(b), there is some discrepancy between the calculated results based on the material parameters in Tables 4 and 5 and the experimental data. However, overall, the deviatoric stress and pore water pressure values of the samples increase with increasing confining pressure. At low confining pressures, there are significant peaks in the deviatoric stress-axial strain and pore water pressure-axial strain curves, indicating the strain softening behavior of the structured soil. As the confining pressure increases, the samples exhibit strain hardening characteristics, which is consistent with the behavior observed in the experimental curves of structured soil.

**Fig 13:**
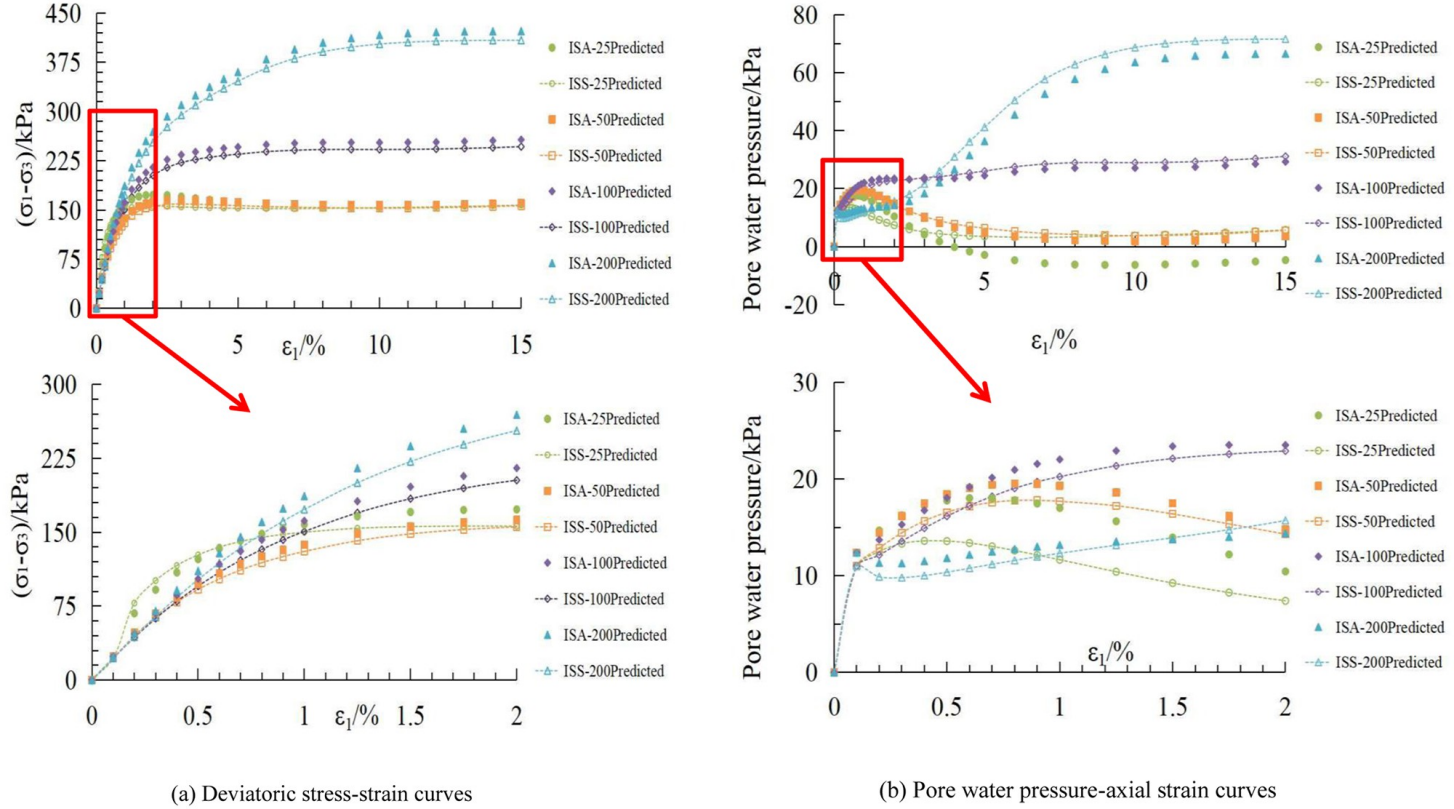
Calculation curves of the two structured soils. (a) Deviatoric stress-strain curves. (b) Pore water pressure-axial strain curves.

Comparison of the deviatoric stress-axis strain and pore water pressure-axis strain curves for the two structured soils obtained through the incremental form of the binary-medium model is shown in Fig 13(a) and 13(b).

The initial stress anisotropic structured soil, due to the lateral rigidity constraint from the applied load and a three-part mold during the curing process, exhibits higher strength in the brittle-elastic components compared to the isotropic structured soil. As a result, the deviatoric stress-strain curve of the initial stress anisotropic structured soil is higher than that of the isotropic structured soil; Under the action of load, the initial stress anisotropic structured soil’s soil skeleton can withstand a higher load. Therefore, its pore water pressure is lower than that of the isotropic structured soil. At lower confining pressures, the structural damage in the structured soil is relatively low. However, as the axial strain increases, shear dilation occurs in the sample, resulting in negative pore water pressure and the occurrence of a peak in the deviator stress-strain curve. With increasing confining pressure, the sample gradually exhibits strain hardening characteristics. The pattern of the curves in Fig 13 is consistent with the experimental curves, demonstrating the same behavior.

## 5 Conclusions

This study focuses on artificially structured soils and conducts consolidated undrained triaxial tests under various confining pressures. With increasing confining pressure, the deviatoric stress and the peak pore water pressure of the three soil samples both show an increasing trend. Due to the differences in structural characteristics among the soil samples, the load-bearing behavior of the soil skeleton varies. Therefore, the initial stress anisotropic structured soil can withstand a greater differential stress compared to isotropic structured soil and remolded soil under the same consolidation stress. However, the variation pattern of pore water pressure is opposite to that of the deviator stress. The results indicate that at lower consolidation stresses, there is less structural damage in the structured soil during the consolidation process. The specimens exhibit significant yield strength, during the experimental process, it is observed that the specimens exhibited negative pore water pressure and experienced shear dilatancy. However, at higher consolidation stresses, all three specimens exhibit strain hardening behavior. Under the influence of the consolidation stresses, there is more structural damage in the structured soil, and its characteristics tend to resemble those of remolded soil.

An incremental form of the binary-medium model is employed to simulate the mechanical features of the artificially structured soils. This model utilizes a linear elastic constitutive model and the Lade-Duncan elastoplastic constitutive model to characterize the stress-strain relationships of the bonded and frictional components in the binary-medium model. The results show that the proposed model can effectively capture the strain-softening and strain-hardening characteristics of the structural soil under both low and high confining pressures. Additionally, the calculated results based on the initial stress anisotropic structured soil and isotropic structured soil can also reflect the influence of structural differences on the stress-strain behavior of the soil. Overall, the calculated results are consistent with the experimental results. In conclusion, the model is able to effectively capture the influence of structure on the stress-strain behavior of the soil.

Besides considering the ununiform distribution of stress and stain within the soil sample, the constitutive model developed here can consider the anisotropy of the structured soil. Furthermore, based on the test results under undrained conditions, we determine the relevant parameters of the incremental form of the binary medium constitutive model and complete the modeling of the experimental results, in which the pore water pressure can be predicted relatively well.
